# Maternal Melatonin Supplementation Modulates Placental DNA Methylation and Gene Expression in Nutrient-Restricted Cattle

**DOI:** 10.3390/ijms262311387

**Published:** 2025-11-25

**Authors:** Shiveeli Rajput, Brittni Littlejohn, Zully E. Contreras-Correa, Hala El Daous, Darcie Sidelinger, Heath King, Mark Arick, Caleb Lemley

**Affiliations:** 1 Department of Animal and Dairy Sciences, Mississippi State University, Mississippi State, MS 39762, USA; sr2166@msstate.edu (S.R.); he226@msstate.edu (H.E.D.); 2 Department of Animal Science, Division of Agriculture, University of Arkansas, Fayetteville, AR 72702, USA; bplitt@uark.edu; 3 Department of Animal Science, Tarleton State University, Stephenville, TX 76402, USA; zcontrerascorrea@tarleton.edu; 4 Faculty of Veterinary Medicine, Benha University, Mushtuhur 13736, Qalyubia, Egypt; 5 Department of Pathobiology and Population Medicine, Mississippi State University College of Veterinary Medicine, Mississippi State, MS 39762, USA; darcie.sidelinger@msstate.edu (D.S.); heath.king@msstate.edu (H.K.); 6 Institute for Genomics, Biocomputing & Biotechnology, Mississippi State University, Mississippi State, MS 39762, USA; maa146@igbb.msstate.edu

**Keywords:** maternal nutrition, melatonin supplementation, placental cotyledons, DNA methylation, transcriptomic profiling, sex-specific differences

## Abstract

This study investigated the influence of maternal nutrient restriction and dietary melatonin supplementation on DNA methylation and gene expression in bovine placental cotyledons, with a focus on sex-specific changes. On day 160 of gestation, 29 Brangus heifers (bred to a single sire by AI) were subjected to a 2 × 2 factorial design: adequately fed (ADQ-CON, n = 7), nutrient-restricted (RES-CON, n = 7), and adequately fed or nutrient-restricted supplemented with 20 mg/d of melatonin (ADQ-MEL, n = 7; RES-MEL, n = 8). Cotyledons were collected at day 240 from 12 female and 17 male conceptuses for Methyl MiniSeq-GWBS and RNA-Seq. In RES-CON vs. ADQ-CON, 93 hypomethylated and 143 hypermethylated DMRs were identified, primarily in exonic, intronic, and promoter regions. Melatonin altered the methylation patterns of male and female cotyledons, respectively, with 203 and 460 DMRs associated with axon guidance, RHOC GTPase cycle, and BDNF signaling pathways. RES-MEL showed higher expression of the *UBOX5* gene compared with RES-CON. Moreover, 15 DEGs (5 upregulated and 10 downregulated) were observed in the male vs. female comparison. In melatonin-treated males, *PIGX*, *ATP11C*, *snoRNA U2-19*, *ZNF82* genes were upregulated. Thus, melatonin may modulate conceptus growth and development in a sex-specific manner.

## 1. Introduction

Maternal nutrition plays a crucial role in fertility and conceptus development during the periconceptual period, with inadequate nutrient intake often leading to impaired fetal development and an increased risk of metabolic diseases later in life [1,2]. In livestock, nutrient restriction during this period negatively affects oocyte quality, which is vital for oocyte fertilization, early embryonic, placental and fetal development during gestation. This, in turn, affects pregnancy outcome and offspring health across various species [3,4,5]. The development of the conceptus during gestation is a result of highly organized, coordinated alterations that are primarily fueled by maternal nutrition [6]. Prenatal malnutrition (i.e., over- or under-nutrition) experienced in utero is a primary contributor associated with adverse health outcomes [7]. Intrauterine growth restriction (IUGR) is one of the most recognized outcomes of poor maternal nutrition [7,8]. IUGR is a condition where the conceptus does not grow as expected, with body weight and length falling below the 10th percentile for its gestational age [9,10]. In humans, approximately 70–80% of IUGR cases are attributed to utero-placental insufficiency, which results from abnormal placental vascular development [11,12]. In ruminants, 60–90% of conceptus growth and development occurs during the third trimester of pregnancy, when placental angiogenesis is critical to sustain the substantial increase in blood flow [13]. When perfusion is reduced, the risk of cardiovascular and neurological diseases in offspring increases [14,15]. These developmental issues can produce long-term alterations in gene expression through epigenetic mechanisms, thereby linking suboptimal conceptus nutrition to lasting health outcomes. Maternal nutrition does not alter the genomic makeup of the offspring; however, it significantly affects the expression of key genes that are regulated through epigenetic mechanisms such as DNA methylation, histone modifications, and non-coding RNAs [16]. One prominent example of such epigenetic regulation is the methylation of the glucocorticoid receptor (GR) gene that has been linked to the sex-specific alterations in glucose metabolism of the offspring [17]. Other studies have shown that maternal under- or over-nutrition during pregnancy can affect the expression of key metabolic genes, such as *WNT5A*, *IGF2*, *RXRA*, *AKT*, and *PI3K*, and subsequent histone modification influences genes like *WNT1*, *PPARα*, *GR*, and *IGF1*, all contributing to long-term changes in the metabolism in the offspring [16]. Despite advancements in therapeutic strategies and close monitoring of high-risk pregnancies, optimal outcomes have not been achieved, highlighting the need for innovative treatment approaches [18]. Melatonin, a neuroendocrine hormone produced by the pineal gland, has emerged as a promising therapeutic agent due to its dual ability to enhance blood flow while also mitigating oxidative stress [19]. Furthermore, melatonin, due to its lipophilic properties, can easily travel through the placenta and enter the fetal circulation unaltered [20,21], and it plays a critical role in regulating circadian rhythms [20,22]. Melatonin directly scavenges reactive oxygen species (ROS) produced from mitochondrial respiratory chain electron leakage and preserves the structural or functional integrity of antioxidative enzymes [23]. It effectively reduces the ROS, which may alleviate conceptus growth restriction caused by oxidative damage during compromised pregnancies [24,25]. Previous studies suggest that melatonin may alleviate some of the adverse effects of nutrient restriction during pregnancy [26]. It has been shown that melatonin can improve developmental outcomes by regulating epigenetic and transcriptomic mechanisms such as DNA methylation and histone modifications, as well as by lowering the oxidative stress and apoptosis, in porcine embryos [27]. Despite these promising functions, the molecular mechanisms through which melatonin influences the bovine placental function and conceptus development remain unclear. Thus, we hypothesize that dietary melatonin supplementation may alleviate comprised mid to late pregnancies through epigenetic and transcriptomic mechanisms. The present study aimed to investigate the effects of maternal nutritional restriction and dietary melatonin supplementation on placental cotyledons’ DNA methylation and gene expression changes, with a particular focus on sex-specific differences.

## 2. Results

### 2.1. Methylome and Transcriptome Profiles

The Methyl MiniSeq genome-wide bisulfite sequencing (Methyl MiniSeq-GWBS) resulted in an average CpG coverage of 11.16 (ranging from 7.21 to 14.37). The RNA Seq analysis generated an average of 48.99 million raw reads per sample (ranging 42.8–59.4). After filtering the raw read data, 48.88 million (99.77%) clean reads were obtained. Approximately 46.97 million (96.09%) of these clean reads were successfully mapped to the *Bos taurus* reference genome (ARS-UCD1.2). The analysis confirmed an average of 56.07% of reads were assigned to the coding DNA sequence regions (CDS), while 7.6% aligned to the untranslated regions (UTRs), 20.57% to introns, 10.9% to the transcription start sites (TSS) and transcription end sites (TES), and 4.86% to other intergenic regions. Detailed information about the sequencing data is given in Table S1.

### 2.2. DNA-Methylation Profiling Across Experimental Groups

#### 2.2.1. Differential DNA Methylation by Nutrient Restriction

We observed 2435 differentially methylated cytosines (DMCs) (*adj. p*-value < 0.05) in the RES-CON vs. ADQ-CON cotyledons. This included 983 hypomethylated (*adj. p*-value < 0.05 and methDiff < −0.1) and 1452 hypermethylated sites (*adj. p*-value and methDiff > −0.1). Additionally, 236 differentially methylated regions (DMRs) were observed between the RES-CON vs. ADQ-CON cotyledons. Of these, 93 DMRs exhibited hypomethylation and 143 showed hypermethylation (Figure S1). The DMRs were predominantly located within the exonic, intronic, and promoter regions. Notably, several differentially methylated genes (DMGs) including *LEFTY2*, *MIR455*, *TNPO2* (LOC112447621) and *COX19* showed methylation in the promoter region (Table S2). The DMRs were predominantly located on Chromosomes 11, 13, 16, 17, 18, 22, 23, and 25. Detailed information about hypomethylated and hypermethylated DMGs are given in Table 1 and Table S2.

#### 2.2.2. Melatonin Dependent Differential DNA Methylation

**Figure 1:**
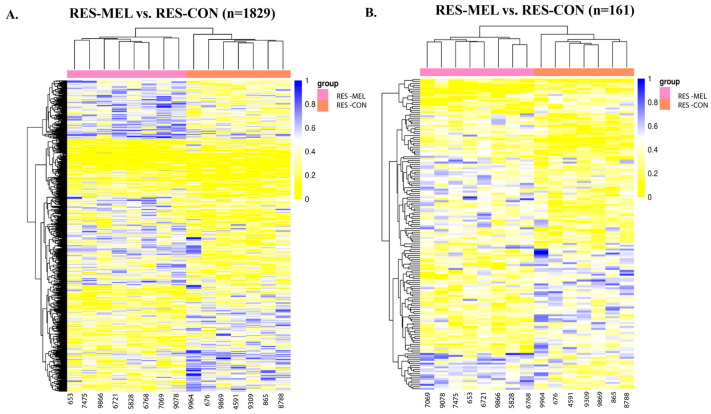
Heatmaps showing the hierarchical clustering of DMCs and DMRs in the genomic regions for RES-MEL vs. RES-CON cotyledons. (**A**) Heatmap showing the distribution of DMCs. There were 779 hypomethylated and 1050 hypermethylated DMCs in the RES-MEL vs. RES-CON cotyledons (*adj. p*-value < 0.05). (**B**) Heatmap showing the distribution of DMRs. There were 45 hypomethylated and 114 hypermethylated regions (*adj. p*-value < 0.05). The rows represent the DMCs and DMRs, and the column denotes the sample. The level of darkness of each color represents the amount of deviation compared to the mean value. Abbreviations: RES—restricted, MEL—melatonin, CON—control group.

To evaluate the melatonin diet-dependent effect on the epigenome, we compared ADQ-MEL vs. ADQ-CON, RES-MEL vs. RES-CON, and RES-MEL vs. ADQ-MEL cotyledons. In the ADQ-MEL vs. ADQ-CON cotyledons, we observed 2385 DMCs (*adj. p*-value < 0.05), with 1149 hypomethylated (*adj. p*-value < 0.05 and methDiff < −0.1) and 1236 hypermethylated sites (*adj. p*-value < 0.05 and methDiff > −0.1) (Figure S2A). In addition, a total of 223 DMRs, with 108 hypomethylated and 115 hypermethylated regions, were determined (Figure S2B). These DMRs were predominantly located in the exonic and the intronic regions, whereas some of the hypermethylated DMGs, such as *C4H7orf25*, *ZCCH13*, and *TADA2B*, and hypomethylated DMGs, such as *LEFTY2*, and *DAG1*, showed methylation in the promoter region (Table S3). In the RES-MEL vs. RES-CON cotyledons, we observed 1829 DMCs encompassing 779 hypomethylated and 1050 hypermethylated sites (Figure 1A). In addition, we observed 161 DMRs, with 71 hypomethylated and 90 hypermethylated regions in RES-MEL compared with the RES-CON group (Figure 1B). The hypomethylated DMGs such as *MIR411A*, *MIR299*, and *CD93* exhibited methylation in the promoter region in MEL-RES vs. CON-RES cotyledons (Table S4). Likewise in RES-MEL vs. ADQ-MEL cotyledons, 1677 DMCs with 475 hypomethylated and 1202 hypermethylated sites were found (Figure S3A), whereas a total of 159 DMRs were found, with 45 hypomethylated and 114 hypermethylated regions (Figure S3B). These DMRs were predominantly located in the exonic, intronic and promoter regions. Detailed information about the top differentially methylated genes across different experimental groups is given in Table 1, and chromosomal location is given in Table S5.

#### 2.2.3. Sex-Specific DNA Methylation Differences

**Figure 2:**
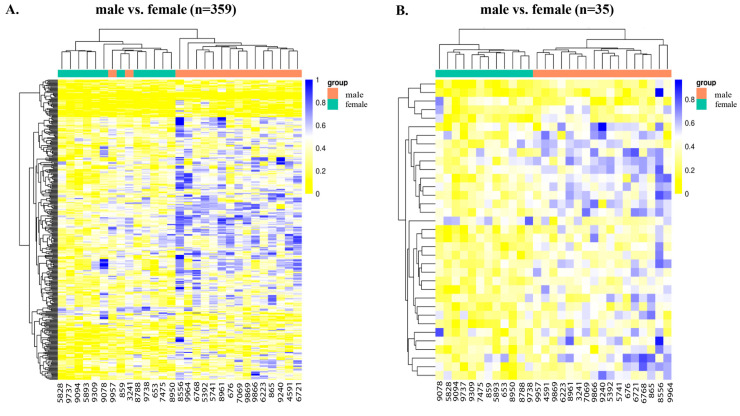
Heatmaps showing the hierarchical clustering of DMCs and DMRs in the genomic regions for male vs. female cotyledons. (**A**) Heatmap showing the distribution of DMCs. There were 22 hypomethylated and 337 hypermethylated DMCs in the male vs. female cotyledons (*adj. p*-value < 0.05). (**B**) Heatmap showing the distribution of DMRs. There were 2 hypo and 33 hyperDMGs (*adj. p*-value < 0.05) in the male vs. female cotyledons. The data are displayed in a grid, where each row represents the DMCs and DMRs, and each column represents the sample. The level of darkness of each color represents the amount of deviation compared to the mean value.

**Figure 3:**
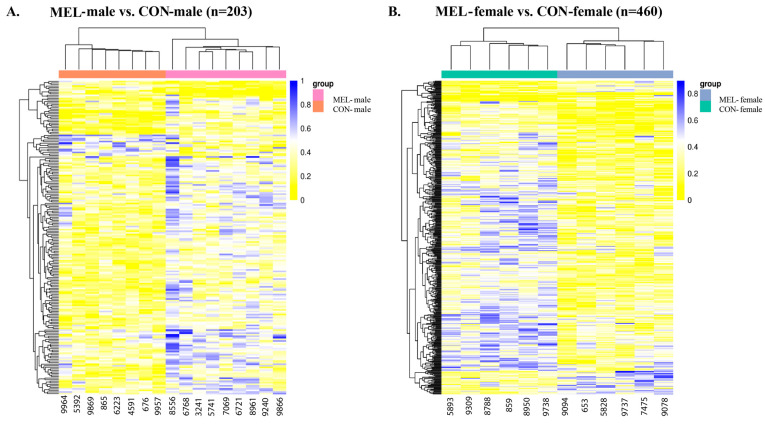
Heatmaps depicting differential methylation in the genomic regions for MEL-male vs. CON-male cotyledons and MEL-female vs. CON-female cotyledons. (**A**) Heatmap showing the distribution of DMRs in the MEL-male vs. CON-male cotyledons. There were 25 hypoDMRs and 178 hyperDMRs (*adj. p*-value < 0.05). (**B**) Heat showing the distribution of differential methylation in the MEL-female vs. CON-female cotyledons. There were 400 hypoDMRs and 60 hyperDMRs. The data are displayed in a grid, where each row represents the DMRs, and each column represents the sample. The level of darkness of each color represents the amount of the deviation compared to the mean value. Abbreviations: MEL—melatonin, CON—control.

To explore the sex-specific epigenetic changes and how melatonin modulates them, we compared the male vs. female cotyledons. A total of 359 DMCs with 22 hypomethylated (*adj. p*-value < 0.05 and methdiff < −0.1) and 337 hypermethylated (*adj. p*-value < 0.05 and methDiff > −0.1) sites were observed (Figure 2A). We also observed 35 DMRs, encompassing 2 hypomethylated and 33 hypermethylated DMGs, in the male compared with female cotyledons (Figure 2B and Table S6). The hypomethylated DMRs were predominantly located in the exonic region of the *ACAD8* and *ZNF532* genes, while hypermethylated DMRs were located in the exonic, intronic, and promoter regions (Table S6). To understand the effect of melatonin on the male and female cotyledons in relation to the control, we compared the MEL-male vs. CON-male cotyledons, and MEL-female vs. CON-female cotyledons. We observed 203 DMRs between the MEL-male vs. CON-male group, encompassing 25 DMRs that exhibited hypomethylation and 178 hypermethylation (Figure 3A and Table S7). Hypermethylation of several genes like *ADAD2*, basic salivary proline-rich protein 1-like (LOC107131929), *ZNF548* (LOC789715), and *NNAT*, and hypomethylation of U6 Spliceosomal protein were observed in the promoter region. Differential methylation analysis in the MEL-female vs. CON-female cotyledons revealed a total of 460 DMRs, with 400 being hypomethylated and 60 hypermethylated (Figure 3B and Table S7). The majority of these DMRs were located in the exons, and introns, followed by the promoter region (Table S7).

### 2.3. Functional Enrichment of DMGs

**Figure 4:**
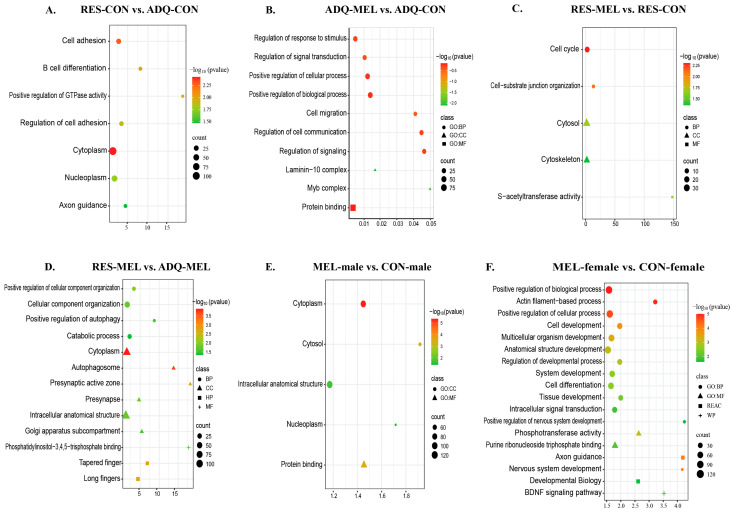
Scatter plot of functional enrichment of genes associated with the differentially methylated regions (DMRs) across different comparisons. Functional enrichment of DMGs: (**A**) RES-CON vs. ADQ-CON cotyledons, (**B**) ADQ-MEL vs. ADQ-CON, (**C**) RES-MEL vs. RES-CON, (**D**) RES-MEL vs. ADQ-MEL, (**E**) MEL-male vs. CON-male, (**F**) MEL-female vs. CON-female. The *y*-axis represents the role in biological processes, molecular function, cellular components and associated pathways; and the *x*-axis represents the fold enrichment (the proportion of DMGs vs. all genes annotated with specific functions). The size of the dots represents the number of genes, and the color represents the −log10(*p*-value). Abbreviations: MEL—melatonin group, CON—control group, RES-MEL—melatonin-supplemented restricted group, RES-CON—restricted control group, ADQ-MEL—melatonin-supplemented adequate group.

To investigate the biological significance of DNA methylation changes, functional enrichment analysis of DMGs was performed using gProfiler to further identify the relevant functions and pathways across six key comparisons. Notably, the RES-CON vs. ADQ-CON comparison revealed functional enrichment in cell-adhesion (GO:0007155), B-cell differentiation (GO:0030183), positive regulation of GTPase activity (GO:0043547), and axon regulation (KEGG:04360), all of which were represented by hypomethylated and hypermethylated DMGs (Figure 4A).

In contrast, ADQ-MEL vs. ADQ-CON was significantly enriched in biological processes such as signal transduction (GO:0009966), cell migration (GO:0016477), cellular communication (GO:0010646), cellular components of the laminin-10 complex (GO:0043259) and Myb complex (GO:0031523) (Figure 4B). In RES-MEL vs. RES-CON, the DMGs were significantly enriched in biological processes such as cell-substrate junction organization (GO:0034330), cellular components of cytosol (GO:0005737|GO:0110165) and molecular functions such as S-transferase activity (GO:0016407|GO:0016417) (Figure 4C). Likewise, the RES-MEL vs. ADQ-MEL comparison revealed enrichment of hypomethylated and hypermethylated DMGs in various biological processes such as positive regulation of autophagy (GO:0010508), catabolic processes (GO:0009056) and cellular components of cytoplasm (GO:0005737), autophagosome (GO:0005776), presynaptic active zone (GO:0048786), presynapse (GO:0098793), intracellular anatomical structure (GO:0005622), and golgi apparatus subcompartment (GO:0098791), as well as molecular functions like phosphatidylinositol-3, 4, 5 triphosphate binding (GO:0005547) (Figure 4D).

To further explore the effect of melatonin on sex-specific conceptus changes, we performed functional enrichment of the DMGs. In the Mel-treated males (MEL-male vs. CON-male), DMGs were predominantly enriched in cytoplasm (GO:0005737), nucleoplasm (GO:0005654), and protein binding functions (GO:0005515) (Figure 4E). Interestingly, the MEL-female vs. CON-female cotyledons revealed the functional enrichment of hypomethylated and hypermethylated DMGs in the cell development (GO:0048468), multicellular organism development (GO:0007275), anatomical structure development (GO:0048856), cell morphogenesis (GO:0000902), macromolecule modification (GO:0043412), neurogenesis (GO:0022008), cytoskeleton organization (GO:0007010), intracellular signal transduction (GO:0035556), regulation of cellular component biogenesis (GO:0044087), and positive regulation of nervous system development (GO:0051962). The hypomethylated DMGs were significantly associated with axon guidance pathways (REAC:R-BTA-422475), Fcgamma receptor (FCGR)-dependent phagocytosis pathways (REAC:R-BTA-2029480), regulation of actin dynamics for phagocytic cup formation (REAC:R-BTA-2029482), RHOC GTPase cycle signaling by Rho GTPases (REAC:R-BTA-9013408), and BDNF signaling pathways (WP:WP3141) (Figure 4F). Comprehensive functional enrichment details are provided in Table S8.

### 2.4. RNA Sequencing Profiles of Differentially Expressed Genes

**Figure 5:**
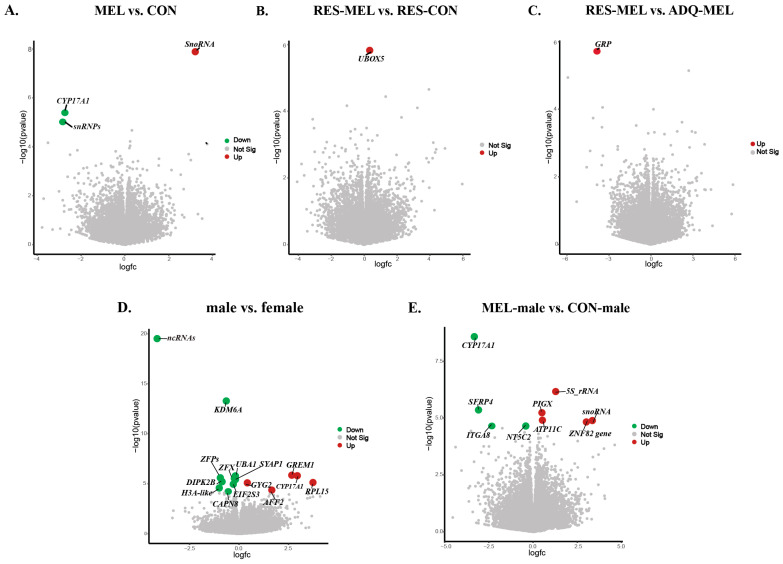
Visualization of statistically significant DEGs (*adj. p*-value ≤ 0.05) across different comparisons. The volcano plot shows the log of fold change (logFC) plotted against -log10 (*p*-value) for DEGs: (**A**) MEL vs. CON cotyledons, (**B**) RES-MEL vs. RES-CON cotyledons, (**C**) RES-MEL vs. ADQ-MEL cotyledons, (**D**) male vs. female cotyledons, and (**E**) MEL-male vs. CON-male cotyledons. The colored dots represent the upregulated and downregulated genes. Green and red represent the upregulated and downregulated genes, respectively. Abbreviations: MEL—melatonin group, CON—control group, RES-MEL—melatonin-supplemented restricted group, RES-CON—restricted control group, ADQ-MEL—melatonin-supplemented adequate group.

DESeq2 analysis identified a total of 23,937 expressed genes; however, in the RES-CON compared with the ADQ-CON cotyledons, we did not observe statistically significant DEGs (*adj. p*-value > 0.05). To explore the effect of melatonin on conceptus gene expression, we compared the melatonin-treated group with the control group (MEL vs. CON cotyledons) and found a total of three significant DEGs based on absolute log2(FC) and *adj. p*-value ≤ 0.05. Among these, the small nucleolar RNA U2-19 genes (*SnoRNA U2-19*) were upregulated and the *CYP17A1* gene and small nuclear RNPs E (*snRNPs E*) were downregulated in the melatonin-treated group compared with the control (Figure 5A). In the RES-MEL vs. RES-CON cotyledons, we found one gene, i.e., *UBOX5*, to be significantly upregulated (*adj. p*-value ≤ 0.05) in the melatonin-restricted cotyledons compared with control-restricted cotyledons (Figure 5B). Likewise, in the RES-MEL vs. ADQ-MEL cotyledons, we found upregulation of the *GRP* gene (*adj. p*-value ≤ 0.05) (Figure 5C). The calculated log2(FC) values of the DEGs ranged from −2.737506917 (ENSBTAG00000054516) to 3.270511 (ENSBTAG00000042358) (Table S9).

To further explore the sex-specific transcriptomic changes and how melatonin modulates the gene expression, we compared the male vs. female cotyledons. DeSeq2 expression analysis indicated significant expression differences, based on absolute log2 fold change (log2(FC) = −0.18~3.73), (*adj. p*-value ≤ 0.05), within the male compared with female cotyledons. A total of 15 DEGs were found, among which 5 genes (*GREM1*, *CYP17A1*, *RPL15*, *GYG2*, and *AFF2*) were upregulated, and 10 genes (*KDM6A*, *UBA1*, *ZFX*, *ZFPs*, *SYAP1*, *DIPK2B*, *EIF2S3*, *CAPN8*, *ncRNA*, and *H3 Histone 3A*-like genes) were downregulated in the male compared with female cotyledons (Figure 5D). To understand the effect of melatonin on sex-specific changes, we compared the MEL-male vs. CON-male cotyledons and MEL-female vs. CON-female cotyledons. In the MEL-male vs. CON-male cotyledons, we observed 10 differentially expressed genes (*adj. p*-value ≤ 0.05), with the upregulation of *5S rRNA*, *PIGX*, *ATP11C*, *snoRNA U2-19*, and *ZNF82* genes, and the downregulation of *CYPA17A1*, *SFRP4*, *NTC5C2*, and *ITGA8* genes in the melatonin-supplemented male compared with the control male (Figure 5E).

**Figure 6:**
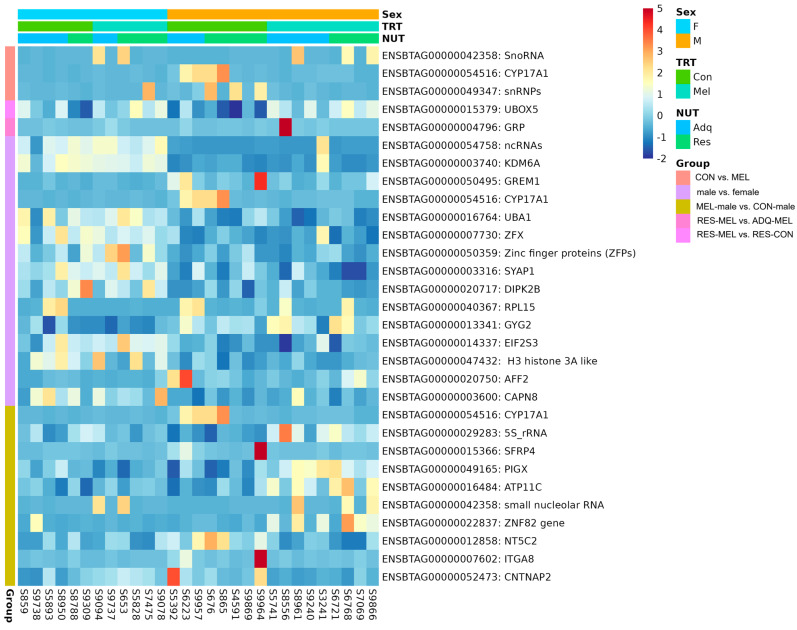
Visualization of statistically significant DEGs (*adj. p*-value ≤ 0.05) across different comparisons. The heatmap shows the hierarchial clustering of DEGs across CON vs. MEL, RES-MEL vs. ADQ-MEL, RES-MEL vs. RES-CON, male vs. female, and MEL-male vs. CON-male cotyledons. The data are displayed in a grid, where each row represents the gene and each column represents the animal sample. The color and intensity of the boxes represents the gene expression levels (upregulated or downregulated genes).

However, we did not observe any significantly differentiated genes in the melatonin-supplemented female cotyledons compared with control female (*adj. p*-value > 0.05). The heatmap showing the significant DEGs in the CON vs. MEL, RES-MEL vs. ADQ-MEL, RES-MEL vs. RES-CON, male vs. female, and MEL-male vs. CON-male cotyledons is shown in Figure 6. The MDS plot is shown in Figure S4. Detailed information about DMGs across different experimental groups is given in Table 2.

### 2.5. Functional Enrichment Analysis of DEGs

To investigate the biological significance of transcriptomic changes, functional enrichment analysis of DEGs was performed using gProfiler across key comparisons. We did not observe significant enrichment of DEGs in the RES-CON vs. ADQ-CON cotyledons (*p* > 0.05). However, the upregulated *UBOX5* gene in the RES-MEL vs. RES-CON cotyledons was shown to be associated with ubiquitin-associated proteolytic pathways (KEGG:04120). Likewise, the upregulated gene GRP in the RES-MEL vs. ADQ- MEL cotyledons was associated with neuroactive ligand receptor interaction (KEGG:04080); peptide hormone metabolism (REAC:R-BTA-2980736); and synthesis, secretion and inactivation of glucagon-like peptide-1 (GLP-1) (REAC:R-BTA-381771). In the male vs. female cotyledons, the DEGs were involved in the negative regulation of osteoclast proliferation (GO:0090291), regulation of peptidyl-tyrosine autophosphorylation (GO:1900084), positive regulation of peptidyl-tyrosine autophosphorylation (GO:1900086), sequestering of BMP from receptor via BMP binding (GO:0038098), regulation of bone trabecula formation (GO:1900154), determination of dorsal/ventral asymmetry, and positive regulation of cell fate commitment (*p* ≤ 0.05) (Figure S5). In addition, the DEGs in the MEL-male vs. CON-male cotyledons were associated with negative regulation of sodium-dependent phosphate transport.

### 2.6. Overlapping Analysis of DNA Methylation and Gene Expression

**Figure 7:**
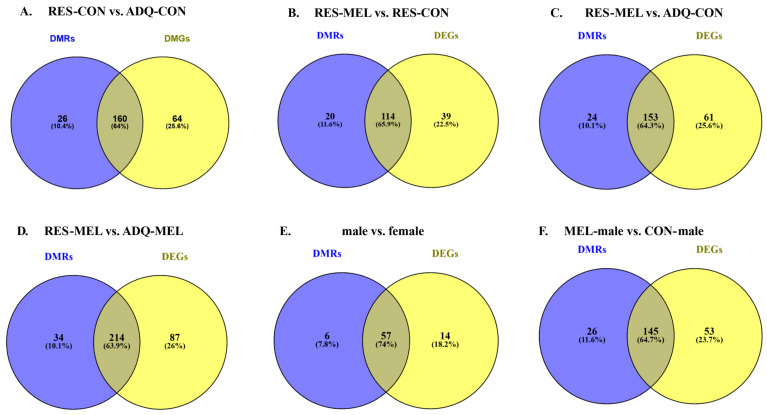
Venn diagram illustrating the number of overlapping genes identified using DNA-methylation and RNA-Seq. Venn diagram shows the overlapping genes across different comparisons: (**A**) RES-CON vs. ADQ-CON cotyledons, (**B**) RES-MEL vs. RES-CON cotyledons, (**C**) RES-MEL vs. ADQ-CON cotyledons, (**D**) RES-MEL vs. ADQ-MEL cotyledons, (**E**) male vs. female cotyledons, and (**F**) MEL-male vs. CON-male. Abbreviations: RES-MEL—melatonin-supplemented restricted group, RES-CON—restricted control group, ADQ-CON—adequate control group, ADQ-MEL—melatonin-supplemented adequate group.

To further explore the genomic effects of melatonin supplementation, we investigated the relationship between DNA methylation and gene expression. Overlap analysis was conducted to identify the overlap genes between the DMRs and DEGs across different comparisons. This analysis identified 160 overlapping genes between RES-CON and ADQ-CON; 114 genes between RES-MEL and RES-CON; 153 overlapping genes between ADQ-MEL and ADQ-CON; and 214 overlapping genes between RES-MEL and ADQ-MEL cotyledons. Additionally, 57 overlapping genes were observed between male vs. female cotyledons. Similarly, a total of 145 overlapping genes were observed in the MEL-male vs. CON-male cotyledons (Figure 7A–F).

The genomic distribution of these overlapping genes was consistent with the results from the Methyl MiniSeq-GWBSanalysis, showing predominant presence in the exonic regions, followed by the intronic, and promoter regions. Notable overlaps at the promoter region included *CD93* in the RES-MEL vs. RES-CON cotyledons; *LEFTY2*, *C4H7orf25*, and *ZCCHC13* in the ADQ-MEL vs. ADQ-CON cotyledons; and *ATG7* and *PLEKHF1* in the RES-MEL vs. ADQ-MEL cotyledons. In addition, promoter region overlaps such as PAWR were identified in the male vs. female cotyledons, and *TAF1C*, *ADAD2*, *CAMK2A* and *MYOM3* in the MEL-male vs. CON-male cotyledons. Detailed information about the overlapping genes is given in Table S10.

## 3. Discussion

Environmental influences such as maternal nutrition during gestation have long-term effects on the developing conceptus via epigenetic and transcriptomic regulation. These impacts, can have a considerable impact on epigenetic programming, altering immune function, fertility, feed intake, and the brain’s reward system, even through transgenerational transmission [28]. The present study investigated the effects of maternal nutrient restriction and dietary melatonin supplementation on the placental methylome and transcriptome, with particular focus on sex-specific differences. The present study supports previous studies showing maternal nutritional restriction can cause congenital defects by limiting nutrient transfer and reducing cellular differentiation, proliferation, and conceptus growth [7,29]. Notably, this study highlights the potential mitigating effects of melatonin supplementation in reducing maternal nutrient restriction-related adverse effects.

### 3.1. Maternal Nutrient Restriction Affects DNA Methylation

The comparison of RES-CON vs. ADQ-CON cotyledons was used to understand the effect of maternal nutrition on the cotyledons. The analysis of DNA methylation patterns revealed varied profiles, with the exonic regions exhibiting higher methylation levels, followed by intronic regions, and promoter regions having lower methylation levels. These showed hypomethylation and hypermethylation in RES-CON compared with ADQ-CON group. It has been reported that DNA methylation affects the accessibility of particular DNA regions for transcription. Specifically, hypomethylation in the promoter region increases the availability of DNA for gene transcription, while hypermethylation reduces transcription [30,31]. Previous studies revealed that maternal nutrition in human, mouse, and sheep models has been shown to alter DNA methylation in a variety of tissue types [32,33,34]. Our findings indicated the significant hypermethylation of multiple genes, including *LEFTY2*, *TNPO2*, *MIR455*, and *COX19* in the promoter region, which are essential for vertebrate embryogenesis [35], nucleocytoplasmic shuttling of developmental proteins [36], and cytosolic transduction [37], in the nutrient-restricted group compared with adequately fed group (RES-CON vs. ADQ-CON). These epigenetic changes can pose long-term effects on the health of offspring. For instance, the left-right determination factor 2 gene (*LEFTY2*), is involved in various biological processes including the left-right asymmetry determination during conceptus development through TGF-β signaling pathways [38]. The hypermethylation of *LEFTY2* in the RES-CON vs. ADQ-CON group indicates that maternal nutrition modulation can impact conceptus development. Goodman and coworkers reported that the alterations in the *TNPO2* gene could also lead to developmental delays, neurological defects, and dysmorphic changes in humans and mice [36]. We did not observe significant gene expression at the transcriptomic level in the RES-CON group when compared with ADQ-CON group.

### 3.2. Melatonin Alters the Epigenetic and Transcriptomic Profile

We further investigated the effects of melatonin supplementation on the epigenome and transcriptome under different dietary conditions in cotyledons. Our findings indicate that melatonin has a diet-dependent effect on the epigenome, causing significant variations in both DMCs and DMRs across different comparisons. Interestingly, we found several CpG-DMCs and DMRs that included both hyper and hypomethylated sites in the ADQ-MEL vs. ADQ-CON, which were enriched in the exonic and intronic regions. These results imply that, when combined with an adequate diet, melatonin may act as a key player in modulating gene expression through epigenetic changes [39]. Interestingly, we observed hypomethylation in the ADQ-MEL groups compared with ADQ-CON group in the promoter region of *LEFTY2*, *DAG1* and several non-coding RNAs, implying the role of melatonin in transcriptional control mechanisms [40]. Previous research has revealed that melatonin impacts DNA methylation by increasing the activity of the ten-eleven translocation (*TET*) enzyme, which promotes DNA methylation and downregulating DNA methyltransferase 1 (*DNMT1*) [39]. This was congruent with our findings where we observed the methylation of *DNMT3A*, indicating its role in cell differentiation and conceptus differentiation [41]. The hypomethylation of the Dystroglycan (*DAG1*) gene in the ADQ-MEL vs. ADQ-CON treatment group was consistent with these reports, suggesting that it performs complex roles at different stages of neural circuit development, including synapse formation, axon guidance, and neuronal migration within the nervous system in the developing conceptus [42]. Similarly, the comparison of RES-MEL vs. RES-CON revealed 1824 CpG DMCs and 161 DMRs, confirming that melatonin’s epigenetic effects persist even under dietary restrictions. Our findings were consistent with previous studies indicating that melatonin affects chromatin remodeling, which in turn, leads to activation or silencing of specific genes by modulating circadian-mediated changes in chromatin structure during the developmental processes [43]. While we observed methylation changes in the promoter, exonic and the intronic regions of several non-coding RNAs, *LEFTY2*, and *DAG1*, we did not identify significant gene expression at the transcriptomic level. The upregulation of the *UBOX5* and *GRP* gene in the RES-MEL vs. RES-CON, and RES-MEL vs. ADQ-MEL groups, suggests that melatonin increases the expression of U-box domain-containing protein (*UBOX5*), which contributes to reducing oxidative stress by degrading proteins that are oxidatively damaged or misfolded during pre-meiotic development of germ cells [44]. Previous reports have shown that *GRP* (gastrin-releasing peptide) expression in the ovine uterus during early pregnancy is correlated with blastocyst growth, formation of a filamentous conceptus and growth and development of the fetus and placenta [45]. Mo C et al. (2025) demonstrated that *GRP* promotes granulosa cell proliferation in chickens by activating the intracellular MAPK/ERK signaling pathway [46].

### 3.3. Sex-Specific Epigenetic and Transcriptomic Alterations

Our findings revealed sex-specific epigenetic and transcriptomic alterations in male and female cotyledons, as well as different responses to melatonin treatment. Interestingly, we discovered hypomethylation of the *ACAD8* and *ZNF532* genes in the male vs. female cotyledons. *ACAD8* encodes an enzyme that is required for the beta-oxidation of mitochondrial fatty acids, which presumably indicates enhanced activity, hence enhancing the amino acid metabolism required for placental growth, angiogenesis and conceptus development [29]. On the other hand, *ZNF532* encodes a transcription factor that is linked to the Stat3 and Wnt/Pcp pathways, which are crucial for regulating the early vertebrate development [47]. We found a total of 15 differentially expressed genes, with 5 upregulated (*GREM1*, *CYP17A1*, *RPL15*, *GYG2*, and *AFF2*) and 10 downregulated (*ncRNAs*, *KDM6A*, *UBA1*, *ZFX*, *ZFPOs*, *SYAP1*, *DIPK2B*, *EIF2S3*, *CAPN8*, and *H3 Histone 3A*-like) genes in the male compared with female cotyledons. The upregulation of *GREM1* in the cotyledons from pregnancies with male fetuses has been shown to induce cell proliferation and embryogenesis, leading to limb development and growth [48]. Previous studies in mice show that upregulation of gremlin leads to the activation of the ERK1/2 pathway, which induces cell proliferation and accumulation of extracellular matrix in mesangial cells [49]. The upregulation of the *CYP17A1* gene in the male conceptus suggests its involvement in the steroid hormone synthesis that may play a critical role in male-specific sex development, differentiation, fertility and the maintenance of hormone balance [50,51]. The *CYP17A1* gene regulates testicular development and spermatogenesis by controlling testosterone synthesis and promoting the proliferation of Leyding cells in goats [52]. Moreover, the upregulation of *AFF2*, *RPL15*, and *GYG2* is shown to be involved in brain development [53], protein synthesis, ribosome development [54], and sex-biased differences during neural development [55]. These results revealed that these genes play an important role in the sex-specific growth and development of the male conceptus. In contrast, these above-mentioned 10 genes were abundantly expressed in female cotyledons. For instance, the *KDM6A* gene encodes a demethylase with female-biased expression that facilitates gene expression by removing the repressive H3K27me3 mark, with evidence of some additional histone demethylase-independent functions [56]. The upregulation of *KDM6A*, *UBA1*, *SYAP1*, and *DIPK2B* genes in female cotyledons compared with male cotyledons suggests a role in sex-specific gene regulation. Previous reports have suggested that some ubiquitination-related genes such as *UBA52* are instrumental to physiological ubiquitination regulation and embryonic development, while modification to this gene can cause early embryonic developmental arrest [57]. Our results on *SYAP1* are consistent with previous findings showing *SYAP1* gene expression and DNA methylation varies between sexes in both myoblasts and myotubes, indicating sex-specific gene regulation [58].

### 3.4. Sex-Specific Epigenetic and Transcriptomic Alterations in Response to Melatonin

The present study revealed that melatonin may modulate the developmental pathways in a sex-specific manner, causing significant variations in both DMRs and DEGs. Interestingly, we observed 203 DMRs that included both hypomethylated and hypermethylated DMGs in the MEL-male vs. CON-male group. HyperDMGs such as *ADAD2*, *ZNF532*, and *NNAT* were associated with several processes including male germ cell development [59], transcriptional regulation [60], and neuronal development [61], while hypomethylated genes such as U6 spliceosomal protein, and *AHDC1* genes have been shown to be involved in regulation of energy metabolism [62]. This suggests that melatonin plays an important role in modulating the biological functions in the male conceptus via epigenetic mechanisms. In contrast, we observed markedly distinct methylation patterns in the female cotyledons in response to melatonin. Our results identified a total of 460 DMRs, with 399 predominantly hypomethylated regions in the MEL-female vs. CON-female cotyledons. The hypomethylated DMGs were significantly associated with axon guidance pathways, regulation of actin dynamics for phagocytic cup formation, RHOC GTPase cycle signaling by Rho GTPases, and BDNF signaling. Axon-guidance pathways regulate the axon guidance and synaptogenesis during early brain development [63]. The majority of axon guidance receptors then act on cytoplasmic proteins to control Rho family small GTPases, which alter cytoskeletal and membrane dynamics via downstream effectors [64,65]. Brain-derived neurotropic factor (BDNF) is crucial for ovarian processes including follicle development, oocyte maturation and embryonic development [66]. Previous studies have shown that addition of exogenous BDNF reverses the ovarian function by promoting cell proliferation in aged mice [66]. These results suggest that melatonin supplementation influences DNA methylation, thereby activating these pathways in a sex-specific manner.

The RNA Seq analysis further confirmed the DNA methylation findings, showing that the MEL-male vs. CON-male group had upregulated *5S rRNA*, *PIGX*, *ATP11C*, *snoRNA*, and *ZNF82* and downregulated *CYP17A1*, *SFRP4*, *NT5C2*, and *ITGA8* genes. *ATP11C* is a member of the P4-type Adenosine triphosphatase (ATPase) family, which are membrane proteins that have key functions in the maintenance of membrane asymmetry at the early stages of conceptus development [67]. Previous investigations in mice revealed that a global ATP11A gene deficiency caused embryonic lethality, with mutant mice dying presumably from heart attack [68]. Placental abnormalities in ATP11A-deficient embryos may cause reduced blood flow, which in turn, affects heart development, resulting in embryonic mortality [69]. In contrast, melatonin supplementation has shown to increase the uterine blood flow during mid to late gestating cattle, thus reversing the placental defects [70]. The upregulation of *ATP11C*, *PIGX*, and *ZNF82* genes in melatonin-supplemented males shows that melatonin promotes male conceptus development by improving the uterine blood flow. Interestingly, we observed the downregulation of *CYP17A1*, a gene involved in androgen biosynthesis, in melatonin-supplemented males. These results were consistent with previous studies, which revealed that the in vivo administration of melatonin in rats may impact the expression of *CYP17A1*, which regulates steroid production pathways [71]. At this point, our study reveals that melatonin may regulate the expression of *CYP17A1* via cyclic AMP response element modulator (*CREM*) [72], and this gene may act locally on male cotyledons function. However, more studies are needed to understand the sex-specific and time-dependent effects of melatonin in cotyledons. Interestingly, despite the hypomethylated DMGs, we found no significant gene expression in female cotyledons in response to melatonin (MEL-female vs. CON-female). This shows that hypomethylation in female cotyledons may not immediately result in transcriptional activation within the timeframe examined but rather may be priming the chromatin for future activation in later developmental stages [39,73].

### 3.5. Integration Analysis Between DMRs and DEGs

The integration analysis showed substantial overlap between the differentially methylated regions (DMRs) and differentially expressed genes (DEGs) in response to melatonin supplementation. Notably, the overlapping gene *CD93*, also known as complement component C1q receptor in the RES-MEL vs. RES-CON, plays an important role in immune system regulation, angiogenesis, cell–cell proliferation and migration [74]. This gene is shown to enhance angiogenesis, and immune evasion in osteosarcoma by activating the PI3K/AKT pathway [75]. Other overlapping genes such as *LEFTY2*, *C4H7orf25*, and *ZCCHC13* in the ADQ-MEL vs. ADQ-CON cotyledons have been linked to cell differentiation, anterior/posterior axis specification [38], reproductive processes [75], embryonic development and spermatogenesis [76]. Furthermore, we identified *ADAD2*, *CAMK2A*, and *MYOM3* genes in melatonin-supplemented males (MEL-male vs. CON-male), which are involved in cell differentiation, spermiogenesis [77], nervous system development, and muscle and growth differentiation [78]. Previous studies revealed that pituitary *CAMK4* is the primary factor in regulating reproductive behavior in 30-month-old cows through calcium signaling, RAF/MAP kinase signaling, and beta-catenin independent WNT signaling [79]. These findings show that melatonin may have sex-specific effects on reproductive and developmental processes. However, it is important to note that, while we found overlapping genes between DMRs and DEGs, they were not significantly expressed (*p* > 0.05) at the transcriptome level. This suggests that methylation may not be sufficient to affect transcription on its own, or it may serve a more subtle regulatory function, such as changing a gene’s responsiveness to future stimuli or modifying chromatin accessibility without directly affecting transcription.

## 4. Materials and Methods

### 4.1. Experimental Design

Animal care and use procedures were approved by Mississippi State University’s Institutional Animal Care and Use Committee (Protocol #17-709). The animal breeding, handling, and treatments used in this study were previously published by Contreras-Correa [13]. Briefly, a total of 29 pregnant Brangus heifers, which were artificially inseminated with a single Angus bull between 27 March and 12 April 2019, were used. All animals were kept under constant environmental settings that met their nutritional needs for early gestation [80]. On day 140 of gestation, the heifers went through a 20-day acclimation period to become acclimated to receiving feed from an American Calan gate system (American Calan, Northwood, NH, USA). On day 160 of gestation, the animals were arranged in a 2 × 2 factorial design. The animals were subjected to four treatment groups: adequately fed (ADQ-CON; 100% National Research Council (NRC) Recommendation, n = 7), nutrient-restricted (RES-CON; 60% NRC Recommendation, n = 7), adequately fed supplemented with 20 mg/d of melatonin (ADQ-MEL, n = 7), and nutrient-restricted with 20 mg/d of melatonin supplementation (RES-MEL, n = 8). Melatonin was top-dressed in the vitamin-mixed grain after being dissolved in 2ml of absolute ethanol at a concentration of 10 mg/mL, while 2ml of absolute ethanol served as the control treatment [13]. The ethanol was left to evaporate at room temperature before feeding [81]. The group with adequately fed diet received 100% of the net energy recommendations for both maternal and fetal growth [80], whereas the nutrient-restricted groups received 60% of the estimated control diet. The data analysis was conducted based on maternal nutrient restriction such as RES-CON vs. ADQ-CON cotyledons; melatonin treatment such as ADQ-MEL vs. ADQ-CON cotyledons, RES-MEL vs. RES-CON cotyledons, and RES-MEL vs. ADQ-MEL cotyledons; and sex-specific comparisons such as male vs. female cotyledons, MEL-male vs. CON-male cotyledons, and MEL-female vs. CON-female cotyledons.

### 4.2. Collection of Samples

Placental sample collections are described by Contreras-Correa [82]. Briefly, on day 240 of gestation, at 80 days post-treatment, each heifer underwent a Caesarean section while standing in the chute after administration of a paravertebral block using 2% lidocaine. The left oblique portion was aseptically prepared, and an incision of 20 cm was made ventral to the paralumbar fossa’s transverse processes. The fetus forelimbs were located and utilized to help exteriorize the gravid uterus through the abdominal incision. Once the fetus was excised, two placentome adjacent to the umbilical cord were collected [82] and separated between fetal cotyledonary villi and caruncular crypts. Placental cotyledonary tissues collected from female (n = 12) and male (n = 17) conceptuses were snap frozen in liquid nitrogen and stored at −80 °C until further used for nucleic acid isolation, RNA sequencing, and DNA methylation analysis.

### 4.3. DNA Methylation Analysis

#### 4.3.1. Methyl MiniSeq Genome-Wide Bisulfite Sequencing and Library Preparation

The Zymo Quick-DNA/RNA Miniprep Plus kit (Cat#D7003) was used to extract genomic DNA from the placental cotyledon tissues, following the manufacturer’s instructions. The purity and concentration of nucleic acids were assessed using Nanodrop (Thermofischer Scientific, Waltham, MA, USA). The samples were processed and analyzed using the Methyl-MiniSeq Service: Genome-wide bisulfite sequencing (Methyl MiniSeq-GWBS) (Zymo Research, Irvine, CA, USA). For library preparation, a total of 500 ng of genomic DNA was sequentially digested using 60 units of TaqαI, followed by 30 units of MspI (NEB), and then purified with DNA Clean & Concentrator™-5 Kit (Zymo Research, Cat# D4003). Adapter ligation was carried out using pre-annealed adapters containing 5′-methyl-cytosine in place of cytosine, as per Illumina’s specified guidelines. Preparative-scale PCR was performed, and the amplified products were purified with the DNA Clean & Concentrator™-5 Kit (Cat# D4003) for sequencing on an Illumina platform.

#### 4.3.2. Sequence Alignments and Data Analysis

Sequence reads from Methyl Mini-Seq libraries were identified using standard Illumina platform calling software, and then raw FASTQ files were processed with TrimGalore (v0.6.4) to remove adapters, filled-in nucleotides, and low-quality bases. FastQC (v0.11.9) was used to assess the trimmed data quality and the overall read distributions. Reads were aligned to the reference genome using Bismark (v0.22.3), and methylation information was extracted with MethylDackel (v0.5.0), which calculated the methylated and unmethylated reads at each CpG site. Each sampled cytosine’s methylation levels were calculated as the number of reads reporting a C divided by the total number of reads reporting a C or T.

#### 4.3.3. Differential Methylation Analysis

A comparative statistical analysis was conducted to identify, annotate, and visualize differential methylated sites and regions using DSS. Low-coverage cytosines were filtered from the analysis, retaining cytosines with a read depth ≥5 in ≥2 samples in any group. DSS was further used to identify differentially methylated cytosines (DMCs) and differentially methylated regions (DMRs), which employs the Wald test and the Bejamini–Hochberg technique to modify *p*-values. Significant DMCs and DMRs have a false discovery rate (FDR) ≤ 0.05 and an absolute methylation difference of at least 0.1. Annotation data from NCBI were used to annotate DMCs and DMRs by overlapping each DMC and DMRs by overlapping them with additional functional regions such as genes, exons, introns, promoters, and CpG islands, using a minimum overlap threshold of 1 base pair. Subsequently, the g:Profiler tool was employed to perform functional enrichment analysis on the genes linked to the DMRs. Linked genes include all genes that overlap with the DMRs.

### 4.4. Total RNA Extraction, Library Construction and RNA Sequencing

The Zymo Quick-DNA/RNA Miniprep Plus kit (Cat#D7003) was used to extract total RNA from the placental cotyledon tissues, following the manufacturer’s instructions. The Nanodrop2000 spectrophotometer (Thermo Scientific, USA) was used to determine the quality and amount of RNA samples. The Zymo-Seq Ribofree Total RNA Library Prep Kit (Cat# R3000) was used to create libraries from total RNA, following the manufacturer’s instructions manual v1.3.0. RNA was later reverse transcribed into cDNA, followed by ribosomal RNA depletion. A partial P7 adapter was then ligated at the 3′ end of the cDNA, followed by second-strand synthesis and a partial P5 adapter ligation at the 5′ end of the resulting double-stranded DNAs. The libraries were then amplified to incorporate the full-length adapters. Successful library construction was then assessed using Agilent’s D1000 ScreenTape Assay (Agilent, Santa Clara, CA, USA) on the TapeStation. RNA-Seq libraries were then sequenced on the Illumina platform to a minimum sequencing depth of 30 million paired-end reads per sample.

### 4.5. Bioinformatic Analysis

The Zymo Research RNA-Seq pipeline was based on nf-core/rnaseq pipeline v1.4.2 (https://github.com/nf-core/rnaseq, accessed on 18 November 2025) [83] and constructed using Nextflow (https://www.nextflow.io/, accessed on 18 November 2025) [84]. In brief, FastQC v0.11.9 (http://www.bioinformatics.babraham.ac.uk/projects/fastqc, accessed on 18 November 2025) was used to assess the quality of raw sequencing. Trim Galore! V0.6.6 (https://www.bioinformatics.babraham.ac.uk/projects/trim_galore, accessed on 18 November 2025) was further used to trim adapter and low-quality sequences. The trimmed reads were then aligned to the reference genome (ARS-UCD1.2) using STAR v2.6.1d (https://github.com/alexdobin/STAR, accessed on 18 November 2025) [85]. SAMtools v1.9 (https://github.com/samtools/samtools, accessed on 18 November 2025) was used to filter and index BAM files [86]. RNA-Seq library quality was assessed using RSeQC v4.0.0 (http://rseqc.sourceforge.net/, accessed on 18 November 2025) [87] and QualiMap v2.2.2-dev (http://qualimap.conesalab.org/, accessed on 18 November 2025) [88]. Duplication rate quality control was further analyzed using dupRadar v1.18.0 (https://bioconductor.org/packages/dupRadar/, accessed on 18 November 2025) [89]. Reads overlapping with exonic regions were assigned to genes using featureCounts v2.0.1 (https://subread.sourceforge.net/, accessed on 18 November 2025) [90]. Annotations for rRNA genes and exons, along with RepeatMasker rRNA tracks from the UCSC Genome Browser, were used for rRNA classification when applicable. Differential gene expression analysis was performed using DESeq2v1.28.0 (https://bioconductor.org/packages/DESeq2/, accessed on 18 November 2025) [91], and functional enrichment analysis was conducted using the g:Profiler python API v1.0.0 (https://biit.cs.ut.ee/gprofiler/gost, accessed on 18 November 2025) [92]. Visualization of quality control and analysis results was carried out using MultiQC v1.9 (https://github.com/ewels/MultiQC, accessed on 18 November 2025). The volcano plots were constructed using Galaxy server (http://usegalaxy.org, accessed on 18 November 2025) [93].

### 4.6. Analysis of Overlapping Genes

To better understand the relationship between the DMGs and DMRs identified using the DNA methylation and RNA-Seq analysis, respectively, shared genes were filtered. The DMCs and DMRs were annotated with the closest expressed gene in the transcriptome dataset using bedtools v2.31.1 (https://doi.org/10.1093/bioinformatics/btq033, accessed on 18 November 2025). The diagrams were made by the Venny 2.1 Online tool [94].

## 5. Conclusions

Maternal nutrient restriction and melatonin supplementation have been shown to have a direct impact on epigenetic and transcriptome regulation in cotyledons, resulting in sex-specific alterations. Maternal nutrient restriction causes differential methylation of genes, resulting in the suppression of *LEFTY2*, *TNPO2*, and *COX19*, all of which are required for embryogenesis, conceptus growth, and development. Melatonin, a therapeutic agent, was shown to rescue nutrient restriction through diet-dependent epigenetic and transcriptomic alterations. In the present study, melatonin induced clear sex-specific epigenetic and transcriptome changes in the cotyledons, promoting the growth and development of male and female conceptuses. Melatonin affected the DNA methylation and expression of *AHDC1*, *ATP11C*, *PIGX*, and *ZNF82* genes in males, which are involved in energy metabolism, placental development, and reproduction. In female cotyledons, we found extensive hypomethylation but no significant changes in gene expression. This shows that hypomethylation may not have instantly translated into transcriptional activation during the timeframe studied. However, further experimental research at different developmental windows and seasons is required to understand the function of methylation and gene expression in sex-specific alterations in response to melatonin supplementation. The results obtained can further be used as markers to determine optimum conceptus growth and development, minimizing disease risk in animal husbandry.

## Figures and Tables

**Table 1:** Top Differentially methylated genes (*Padj* < 0.05) across different experimental groups in placental cotyledons.

Group	Methylation Change	Adjusted *p*-Value	Top Differentially Methylated Genes
RES-CON vs. ADQ-CON	Hypomethylated	*padj* < 0.05	*PLEKHM2*, *KLF15*, *MCM2*, *SLC22A23*, *ZNF691*, *ITFG2*, *TNPO2*, *HCFC1*, *FLNA*
	Hypermethylated		*LEFTY2*, *COX19*, *COL27A*, *MIR455*, *CHAF1B*, *U2AF2*, *KDM8*
ADQ-MEL vs. ADQ-CON	Hypomethylated	*padj* < 0.05	*CUL4A*, *INPP4A*, *TSNARE1*, *LEFTY2*, *DAG1*, *KDM1B*, *LAMA5*, *APMAP*, *PLEKHM1*, *MSANTD1*
	Hypermethylated		*DNMT3A*, *CHD7*, *CHCD5*, *MYBL2*, *HDAC4*, *LAMB1*, *FLNA*, *ZCCHC13*
RES-MEL vs. RES-CON	Hypomethylated	*padj* < 0.05	*WNT7B*, *NRDE2*, *CD93*, *MIR411A*, *MIR299*, *BCL2*, *ADARB1*, *AHDC1*, *ZNF275*
	Hypermethylated		*EZH1*, *PIK3R1*, *PLCL2*, *CUL4A*, *SPATA13*, *TPRA1*, *HCFC1*, *LAMP1*
RES-MEL vs. ADQ-MEL	Hypomethylated	*padj* < 0.05	*ADAP1*, *PLXND1*, *HDAC7*, *SL12A7*, *PXDN*, *LAMP1*, *MAML3*, *MAD1L1*, *KCTD7*, *MYO10*
	Hypermethylated		*OVOL1*, *ATG7*, *CYP26C1*, *LAMP1*, *IRF8*, *CEPI12*, *ATP9*, *ITBCID5*, *KIF26A*
Male vs. female	Hypomethylated	*padj* < 0.05	*ACAD8*, *ZNF532*
	Hypermethylated		*CAMSAP1*, *MAP2K4*, *TBCID13*, *EZH1*, *PLEKHA6*, *ZNF687*, *PAWR*, *ASTN2*, *LMNB2*
MEL-male vs. CON-male	Hypomethylated	*padj* < 0.05	*BOP1*, *TPRA1*, *PKD1*, *USP12*, *EIPR1*, *FGF14*, *AP1B1*, *FGF18*, *AHDC1*, *MAD1L1*
	Hypermethylated		*CUL1*, *LRP5*, *MED15*, *HDAC4*, *PI4KA*, *NNAT*, *ADAD2*, *TAFIC*, *MYO18A*, *CAMK2A*, *MYOM3*
MEL-female vs. CON-female	Hypomethylated	*padj* < 0.05	*GREB1L*, *EEFSEC*, *ZBED4*, *ADARB1*, *HSF5*, *SLC22A23*, *PYGM*, *TMEM185B*, *TRIM10*
	Hypermethylated		*RREB1*, *KIF25*, *MIR2309*, *LETM1*, *OSGIN1*, *CNST*

**Table 2:** Significant differentially expressed genes (*padj* ≤ 0.05) among different experimental groups in placental cotyledons.

Group	Gene ID	Gene Name	log2FoldChange	*padj*	Gene Expression
MEL vs. CON	ENSBTAG00000042358	*SnoRNA*	3.270511	0.000273	Upregulated
	ENSBTAG00000054516	*CYP17A1*	−2.73751	0.049874	Downregulated
RES-MEL vs. RES-CON	ENSBTAG00000015379	*UBOX5*	0.334699	0.035745	Upregulated
RES-MEL vs. ADQ-MEL	ENSBTAG00000004796	*GRP*	−3.85346	0.042907	Downregulated
Male vs. female	ENSBTAG00000054758	*ncRNAs*	−4.13781	4.77 × 10^−16^	Downregulated
	ENSBTAG00000003740	*KDM6A*	−0.64488	4.2 × 10^−10^	Downregulated
	ENSBTAG00000050495	*GREM1*	2.662567	0.005307	Upregulated
	ENSBTAG00000054516	*CYP17A1*	2.942639	0.005307	Upregulated
	ENSBTAG00000016764	*UBA1*	−0.18675	0.005307	Downregulated
	ENSBTAG00000007730	*ZFX*	−0.23691	0.007499	Downregulated
	ENSBTAG00000050359	*ZFPs*	−0.87648	0.007499	Downregulated
	ENSBTAG00000003316	*SYAP1*	−0.18178	0.007499	Downregulated
	ENSBTAG00000020717	*DIPK2B*	−0.85649	0.011048	Downregulated
	ENSBTAG00000040367	*RPL15*	3.733833	0.011802	Upregulated
	ENSBTAG00000013341	*GYG2*	0.41884	0.011802	Upregulated
	ENSBTAG00000014337	*EIF2S3*	−0.29056	0.014704	Downregulated
	ENSBTAG00000047432	*H3A-like*	−1.00082	0.032569	Downregulated
	ENSBTAG00000020750	*AFF2*	1.655051	0.049011	Upregulated
	ENSBTAG00000003600	*CAPN8*	−0.54618	0.050001	Downregulated
MEL-male vs. CON-male	ENSBTAG00000054516	*CYP17A1*	−3.32335	5.04 × 10^−5^	Downregulated
	ENSBTAG00000029283	*5S_rRNA*	1.278478	0.006595	Upregulated
	ENSBTAG00000015366	*SFRP4*	−3.08592	0.028455	Downregulated
	ENSBTAG00000049165	*PIGX*	0.499777	0.028455	Upregulated
	ENSBTAG00000016484	*ATP11C*	0.527126	0.041514	Upregulated
	ENSBTAG00000042358	*snoRNA*	3.351421	0.041514	Upregulated
	ENSBTAG00000022837	*ZNF82*	3.011589	0.041514	Upregulated
	ENSBTAG00000012858	*NT5C2*	−0.41284	0.049942	Downregulated
	ENSBTAG00000007602	*ITGA8*	−2.33427	0.049942	Downregulated
	ENSBTAG00000052473	*CNTNAP2*	−1.74072	0.054788	Downregulated

## Data Availability

The original contributions presented in this study are included in the article/Supplementary Material. Further inquiries can be directed to the corresponding authors.

## Supplementary Materials

The following supporting information can be downloaded at https://www.mdpi.com/article/10.3390/ijms262311387/s1.
