# Supplementary material for: Maternal Melatonin Supplementation Modulates Placental DNA Methylation and Gene Expression in Nutrient-Restricted Cattle
Source: Int J Mol Sci. 2025 Nov 25;26(23):11387. doi: 10.3390/ijms262311387 (PMC12691978; doi:10.3390/ijms262311387)
Supplement: Supplementary file 1 [file ijms-26-11387-s001.zip › Supplementary figures.pdf]

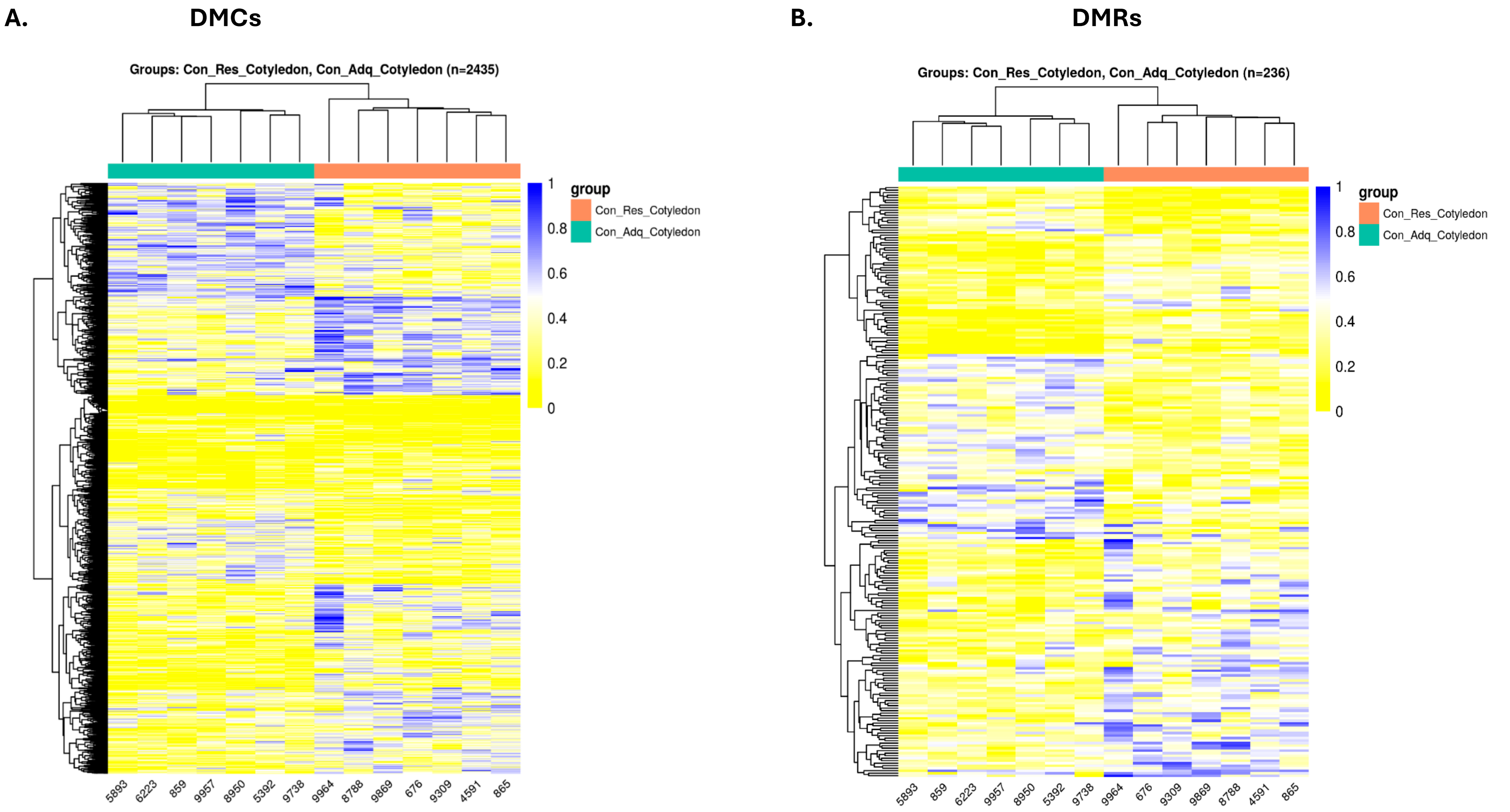

Figure S1. Heatmap showing the hierarchial clustering of differentially methylated cytosines (DMCs) and differentially methylated regions (DMRs) in the genomic regions for Con-Res vs. Con-Adq cotyledons. A: Heatmap showing the distribution of DMCs. There were 983 hypomethylated and 1452 hypermethylated DMCs in the Mel-Res vs. Con-Res cotyledons (padj < 0.05). **B.** Heatmap showing the distribution of DMRs. There were 93 hypomethylated and 143 hypermethylated regions (padj < 0.05). The rows represent the DMCs and DMRs and the column denotes the sample. The darkness of each color represents the magnitude of the difference vs. the mean value.

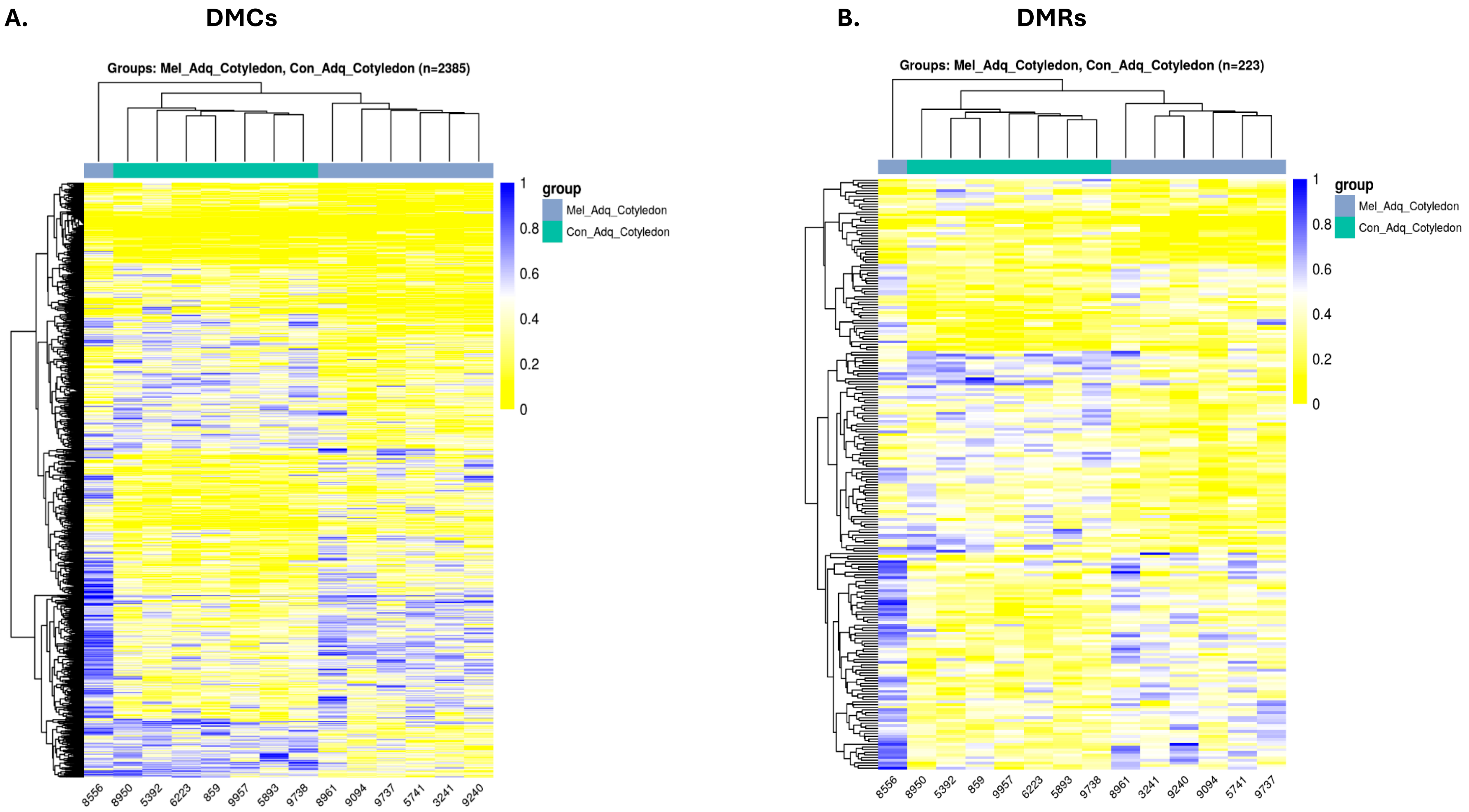

Figure S2. Heatmap showing the hierarchial clustering of differentially methylated cytosines (DMCs) and differentially methylated regions (DMRs) in the genomic regions for Mel-Adq vs. Con-Adq cotyledons. A: Heatmap showing the distribution of DMCs. There were 1149 hypomethylated and 1236 hypermethylated DMCs in the Mel-Adq vs. Con-Adq cotyledons (padj < 0.05). **B.** Heatmap showing the distribution of DMRs. There were 107 hypomethylated and 115 hypermethylated regions (padj < 0.05). The rows represent the DMCs and DMRs and the column denotes the sample. The darkness of each color represents the magnitude of the difference vs. the mean value.

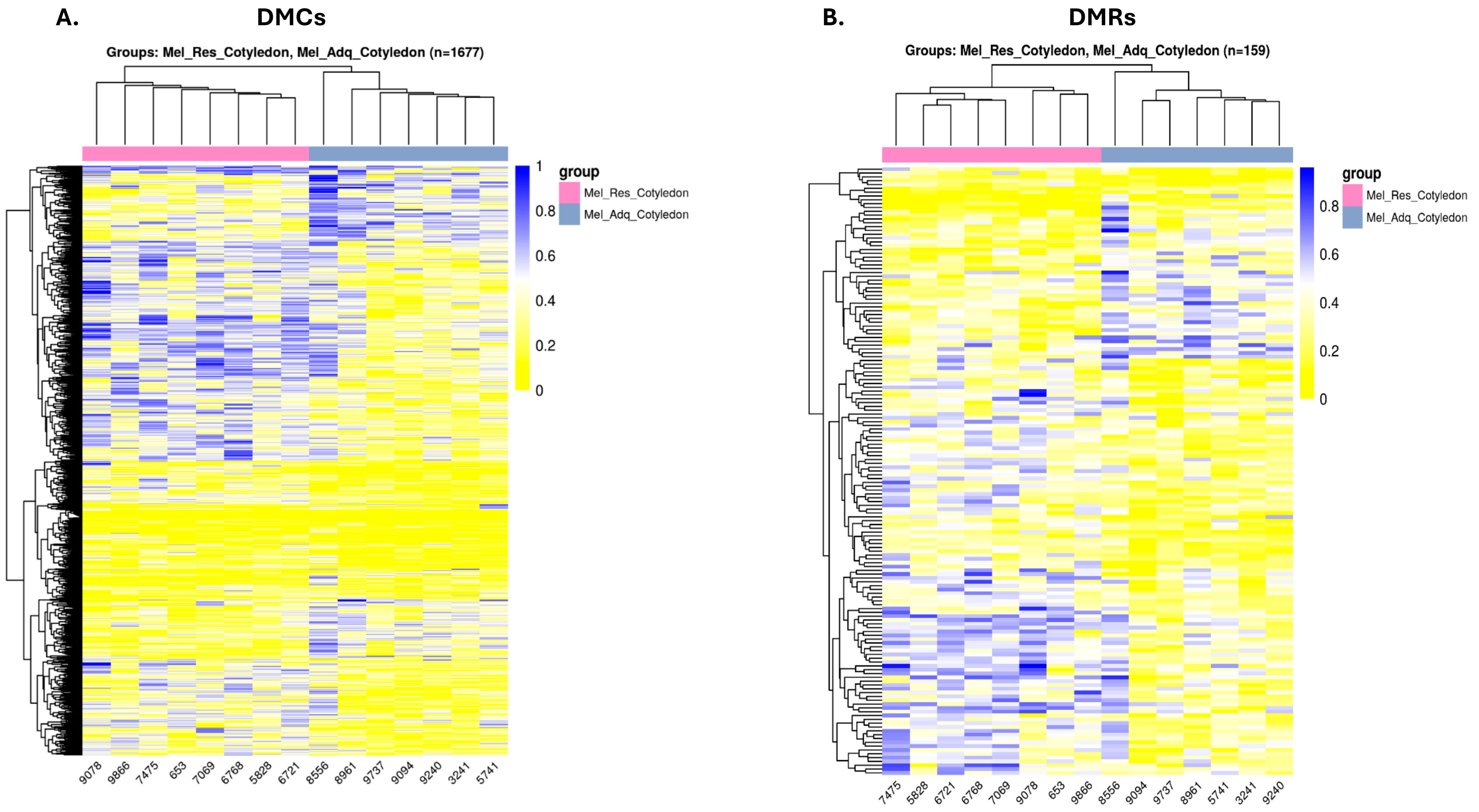

Figure S3. Heatmap showing the hierarchial clustering of differentially methylated cytosines (DMCs) and differentially methylated regions (DMRs) in the genomic regions for Mel-Res vs. Mel-Adq cotyledons. A: Heatmap showing the distribution of DMCs. There were 475 hypomethylated and 1202 hypermethylated DMCs in the Mel-Res vs. Mel-Adq cotyledons (padj < 0.05). **B.** Heatmap showing the distribution of DMRs. There were 45 hypomethylated and 114 hypermethylated regions (padj < 0.05). The rows represent the DMCs and DMRs and the column denotes the sample. The darkness of each color represents the magnitude of the difference vs. the mean value.

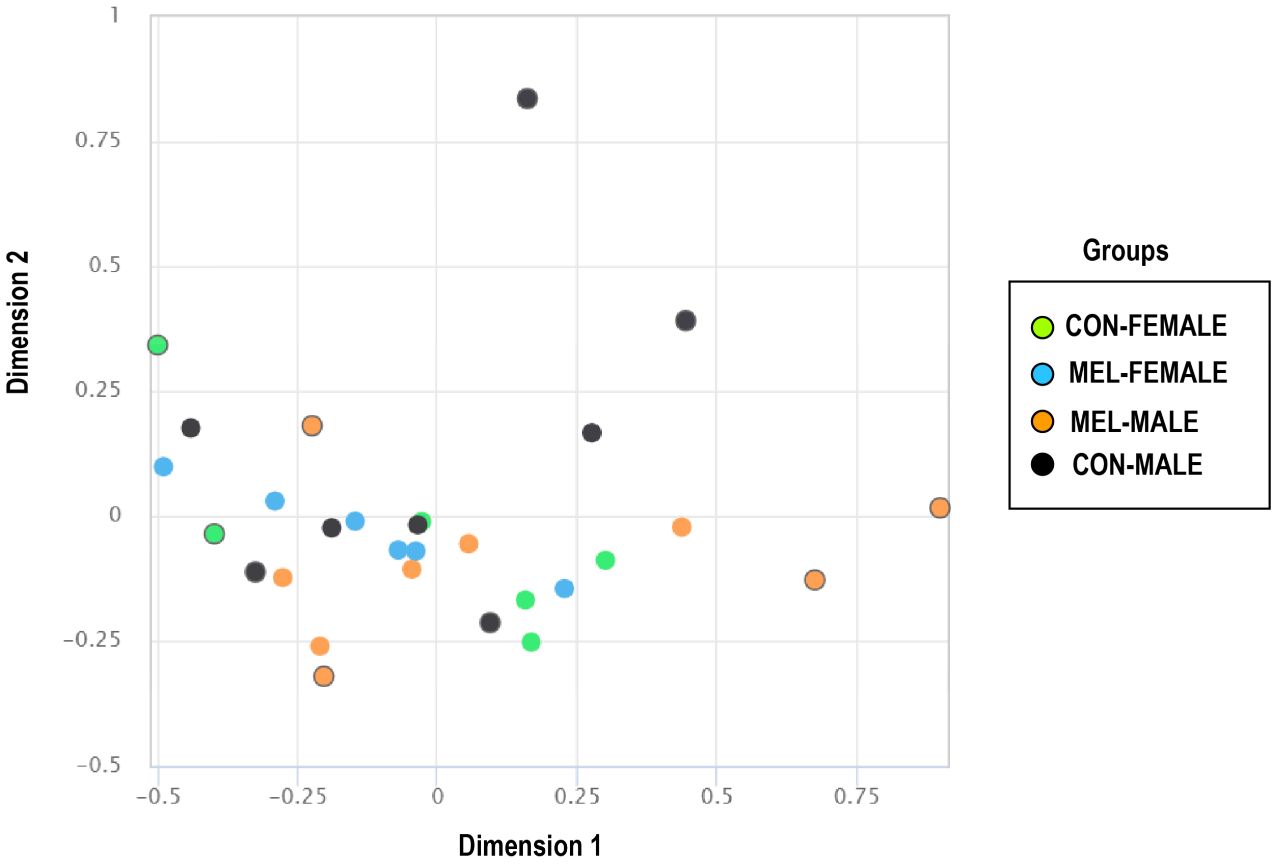

Figure S4: MDS Plot. Multidimensional scaling was conducted to visualize the distance/similarity between samples. Top 500 genes with highest variance among samples were used to make this plot.

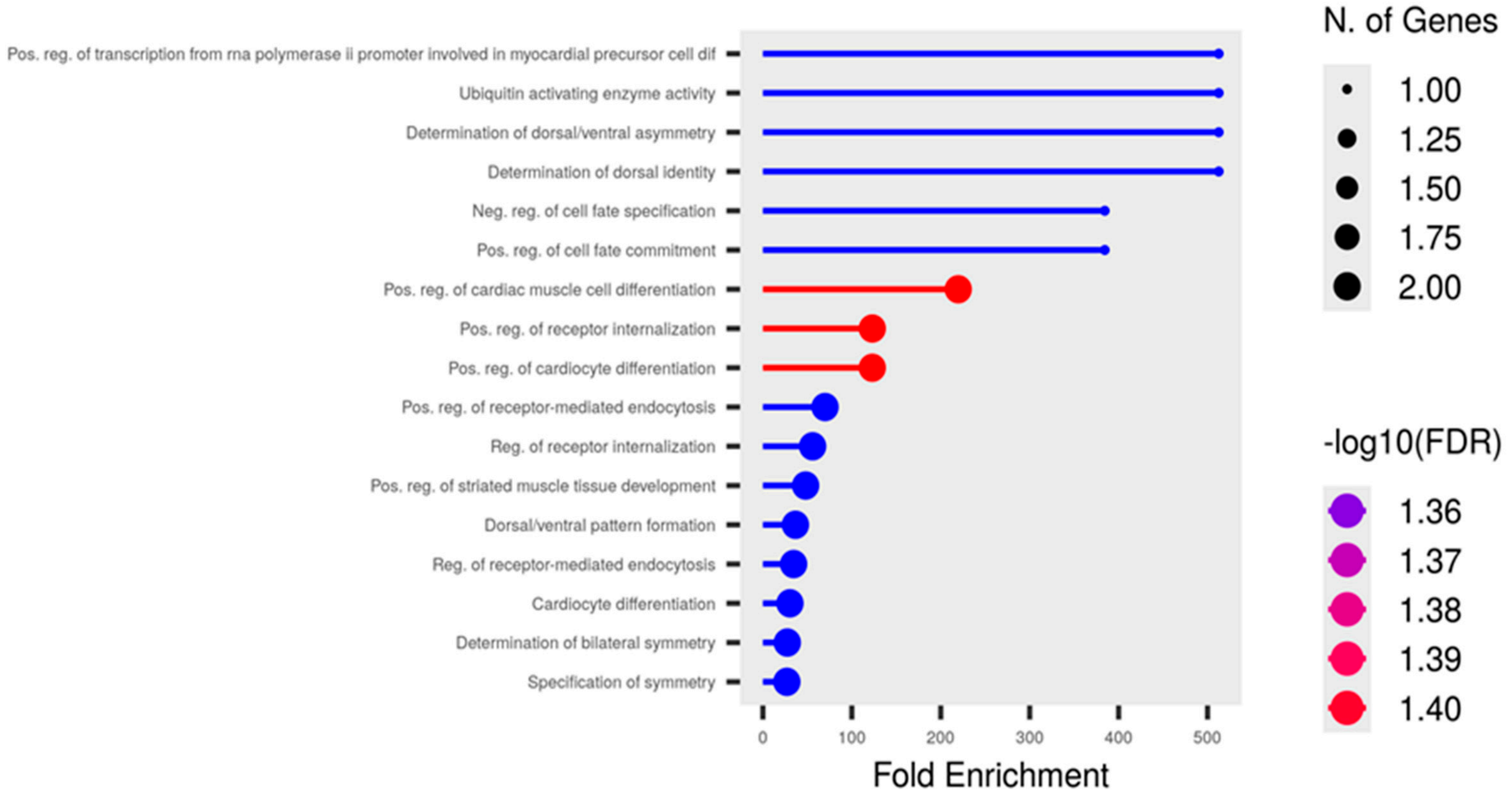

Figure S5. Summary of enriched Gene Ontology (GO) components in MALE vs. FEMALE cotyledons. The dot plot represents results based on the differentially expressed genes (DEGs) in enrichment in the ontology terms. The color represents statistical significance. The Y-axis corresponds to enriched GO terms and the X-axis represents fold enrichment (the proportion of differentially expressed genes (DEGs) vs. all the genes annotated with GO terms. The size of the dot represents the number of genes annotated to GO terms and the color represents the -log10(FDR).
