# Supplementary material for: Maternal Melatonin Supplementation Modulates Placental DNA Methylation and Gene Expression in Nutrient-Restricted Cattle
Source: Int J Mol Sci. 2025 Nov 25;26(23):11387. doi: 10.3390/ijms262311387 (PMC12691978; doi:10.3390/ijms262311387)
Supplement: Supplementary file 1 [file ijms-26-11387-s001.zip › Table S8.pdf]

Group	Functional Enrichment	p-value	Differentially methylated genes (DMGs)
**RES-CON vs. ADQ-CON**	Cell adhesion	0.005081914	SH2B3,KLHL25,COL6A3,PTK2,ABL1,LRFN3,CELSR1,PTPRF,BCR,RREB1,AFDN,PLXNA2,MAD1L1,PLXNA1,DLG5,EMILIN2,BCL2,FES,CARD11,ASS1,SDK1,PTPRJ,FLNA,CDH23,COL26A1
	B cell Differentiation	0.010102247	IRF8,ABL1,EZH2,ITFG2,BCL2,CARD11,PLCG2,PLCL2
	Positive regulation of GTPase activity	0.010223211	BCR,ARAP1,RALGAPA2,TSC2,TBC1D2
	Regulation of cell adhesion	0.013425819	SH2B3,KLHL25,PTK2,ABL1,RREB1,PLXNA2,MAD1L1,PLXNA1,DLG5,EMILIN2,BCL2,FES,CARD11, ASS1,PTPRJ,FLNA,COL26A1
	Cytoplasm	0.003794292	IRF8,IPO9,CHERP,PI4KA,PPP1R12B,KLHL25,UBR4,CARS2,TTC7B,GALNT2,MFHAS1,PLEKHM1,SMO,LIG1 PTK2,ABL1,NAP1L4,RRBP1,TRAPPC9,PTPRN2,RGS11,RGS12,OSBPL10,DGAT1,ARHGEF7,SH3GLB2,BCR, SCYL1,WWOX,ATXN10,ARAP1,KCTD5,NAA60,MRPS25,ABHD12,APBB2,EIPR1,AFDN,DYNC1H1,RNF144A PTPN14,IKBKG,RALGAPA2,PYGB,HTT,QSOX2,MAD1L1,LPAR3,PEX14,ARHGAP39,DGKZ,TSC2,CUX1,EV[illegible] ITFG2,TINAGL1,PLXNA1,SNX25,DLG5,TNPO2,KMT2C,BCL2,UNKL,VAC14,FES,GMDS,HHAT,CARD11, SELENOO,CIB2,PRSS50,RBM18,MCM2,SPTAN1,TBC1D2,MBD1,COX19,MTA1,ASS1,GTF2IRD1,GRK2, RPS6KC1,PDXDC1,EXOSC4,CTBP2,KDM8,FLNA,MVB12B,PES1,DNAH17,CYB5R2,PLCG2,RPS6KA2,RBM1[illegible] IGF2R,FARS2,TPCN1,PYGM,PELP1,PLEKHM2,CASZ1,PYCR3,MRPL13,TFEB,HCFC1,COL26A1,MIPEP
	Nucleoplasm	0.016817889	IRF8,UBR4,CHAF1B,LIG1,TBL1X,ABL1,NCAPD3,CELSR1,RGS12,EZH2,SH3GLB2,ARAP1,RREB1,AFDN,BRD1,PTPN14,HTT,QSOX2,PRPF6,KLF15,DGKZ,CUX1,ITFG2,PLXNA1,KMT2C,RBM18,MCM2,TBC1D2,NRDE2,MTA1,ASS1,GTF2IRD1,EHMT1,EXOSC4,KDM8,PES1,SATB1,UHRF1,RPS6KA2,RBM19,ZNF423,PELP1,CASZ1,U2AF2,RAI1,HCFC1
	Axon guidance	0.034129661	SMO,PTK2,ABL1,PLXNA2,PLXNA1,FES,PLCG2,UNC5A
	bta-miR-23a	0.049980008	ZNF423
	Regulation of response to stimulus	0.004794187	ZNRF3,CHERP,PLCL2,HDAC4,CSNK1E,ING5,FAM53B,RREB1,ENHO,TRAF3IP1,PLCG2,DAGLB,KANK2,WWOX,FLNA,STK24,CTTN,NR1H2,CCAR2,JAG2,GRAMD4,BCR,WWC1,CUL4A,GDF6,CDK3,EMILIN2,MFHAS1,CREBBP,MADD,RPH3AL,STX1A,CRBN,PTK7,SIPA1L3,PRKDC,NPRL2,ITFG2,FCHO1,HTT,NUDT16L1, MEGF8,ZDHHC18,SH3RF3,MYO9B,SKI,SMARCB1
	Regulation of signal transduction	0.010434324	ZNRF3,CHERP,PLCL2,CSNK1E,ING5,FAM53B,ENHO,PLCG2,KANK2,WWOX,FLNA,CTTN,CCAR2,JAG2,GRAMD4,BCR,WWC1,CUL4A,GDF6,CDK3,MFHAS1,CREBBP,MADD,RPH3AL,STX1A,CRBN,PTK7,SIPA1L3, PRKDC,NPRL2,ITFG2,HTT,MEGF8,ZDHHC18,SH3RF3,MYO9B,SKI,SMARCB1

**ADQ-MEL vs. ADQ-CON**	Positive regulation of cellular process	0.012214384	WDR1,CHERP,DYNC1H1,HDAC4,CSNK1E,SLC6A6,PLEKHM1,OSR2,RCC2,ING5,SLC45A3,FAM53B,RREB1,ENHO,CEMIP,BICRA,PLCG2,DAGLB,WWOX,LPIN3,FLNA,MSANTD1,MYO18A,CTTN,NR1H2,MOB3A,MAML3,CCAR2,GAB2,JAG2,BCR,WWC1,CUL4A,MYBL2,MTA1,GDF6,EMILIN2,MFHAS1,CREBBP,RPH3AL,STX1A,CRBN,EVL,PTK7,PRKDC,LAMB1,CTIF,KDM1B,DHX34,FCHO1,STRADA,HTT,MEGF8,WASF2,CHD7,SCARB1,PTMA,SH3RF3,MLXIP,DKC1,SMARCB1
	Positive regulation of biological process	0.013853505	WDR1,CHERP,DYNC1H1,PLCL2,HDAC4,CSNK1E,SLC6A6,PLEKHM1,OSR2,RCC2,ING5,SLC45A3,FAM53B,RREB1,ENHO,CEMIP,BICRA,PLCG2,DAGLB,WWOX,LPIN3,FLNA,MSANTD1,ARIH2,MYO18A,CTTN,NR1H2,MOB3A,MAML3,CCAR2,GAB2,JAG2,GRAMD4,BCR,WWC1,CUL4A,MYBL2,MTA1,GDF6,EMILIN2,MFHAS1,CREBBP,RPH3AL,STX1A,CRBN,EVL,PTK7,PRKDC,LAMB1,CTIF,KDM1B,DHX34,FCHO1,STRADA,HTT,MEGF8,WASF2,CHD7,SCARB1,ZDHHC18,PTMA,SH3RF3,MLXIP,DKC1,SMARCB1
	Cell migration	0.041097363	WDR1,FKRP,RCC2,LAMA5,RREB1,CEMIP,PLCG2,KANK2,FLNA,MYO18A,STK24,ARPC5L,CTTN,BCR,WWC1,EMILIN2,NAV1,EVL,PTK7,LAMB1,MEGF8,WASF2,SCARB1,TNS1
	Regulation of cell communication	0.044806762	ZNRF3,CHERP,PLCL2,CSNK1E,SLC6A6,ING5,FAM53B,ENHO,PLCG2,KANK2,WWOX,FLNA,CTTN,CCAR2,JAG2,GRAMD4,BCR,WWC1,CUL4A,GDF6,CDK3,MFHAS1,CREBBP,MADD,RPH3AL,STX1A,CRBN,PTK7,SIPA1L3,PRKDC,NPRL2,ITFG2,HTT,MEGF8,CHD7,ZDHHC18,SH3RF3,MYO9B,SKI,SMARCB1
	Regulation of signaling	0.046443322	ZNRF3,CHERP,PLCL2,CSNK1E,SLC6A6,ING5,FAM53B,ENHO,PLCG2,KANK2,WWOX,FLNA,CTTN,CCAR2,JAG2,GRAMD4,BCR,WWC1,CUL4A,GDF6,CDK3,MFHAS1,CREBBP,MADD,RPH3AL,STX1A,CRBN,PTK7,SIPA1L3,PRKDC,NPRL2,ITFG2,HTT,MEGF8,CHD7,ZDHHC18,SH3RF3,MYO9B,SKI,SMARCB1
	Laminin-10 complex	0.016746571	LAMA5,LAMB1
	Myb complex	0.049977182	FLNA,MYBL2
	Protein binding	0.003287165	KLHL35,WDR1,LEFTY2,ZNRF3,CHERP,FKRP,DYNC1H1,PLCL2,HDAC4,ESPNL,GMDS,UNC45A,SNX8,RCC2,LRFN3,EXD2,ING5,AGAP1,SLC12A7,LAMA5,SNED1,ENHO,TRAF3IP1,CEMIP,CANT1,LRPAP1,PLCG2,KANK2,SIVA1,WWOX,FLNA,TENM2,THAP4,TPCN2,NOM1,ARIH2,PPP1R37,MYO18A,ARPC5L,CTTN,H2B,TTC7B,NR1H2,BRF1,SYN2,CCAR2,GAB2,JAG2,PRKCZ,WWC1,CUL4A,MTA1,NAP1L4,GDF6,CDK3,EMILIN2,MFHAS1,CREBBP,RPH3AL,STX1A,EVL,SIPA1L3,PRKDC,LAMB1,PHC2,CTIF,ITFG2,KDM1B,TMTC4,WDR20,VTI1A,HTT,SH3TC1,ANKS3,LOC112443175,MAP3K9,MEGF8,WASF2,CHD7,SCARB1,MYO19,PPP1R12B,PTMA,TNS1,DNMT3A,SH3RF3,XPO6,MLXIP,MYO9B,SLC25A13,L3MBTL2,ACOT7,SKI,SMARCB1
	Cell cycle	0.004916271	BCR,TUBGCP3,LLGL2,ADARB1,TRAPPC12,PLEC,HSF1,ERCC6,RCC2,RUVBL1,CNTROB,HTT,BCL2,MAD1L1,CDC45,PKD1,NRDE2,GRK5,CSNK1D,CDT1,TPPP,DYRK3,CUL4A

**RES-MEL vs. RES-CON**	Cell-substrate junction organization	0.007754446	BCR,PLEC,RCC2,BCL2,PIK3R1,ACTN1
	Cytosol	0.02093432	BCR,LAMP1,TUBGCP3,LLGL2,ANKS1A,ADARB1,TELO2,PLEC,PTPN23,HSF1,SEC16A,PITPNM1,FRMD8,GLYCTK,RUVBL1,HTT,HDAC4,ACACA,LIPE,BCL2,PFKP,FASN,PIK3R1,MICAL3,GRK5,ESYT2,THAP4,SLC13A5,ACO2,ZNF746,TTC7B,TBC1D14,TPPP,DYRK3,SPATA13
	Cytoskeleton	0.043985242	PPP1R12B,TUBGCP3,LLGL2,CEP112,PLEC,PTPN23,HSF1,FRMD8,RCC2,CNTROB,HTT,HDAC4,ACACA,ARHGAP39,KIF25,MAD1L1,CDC45,PTK2,CSNK1D,ODF2,ABLIM3,TPPP,DYRK3,ACTN1
	S-acetyltransferase activity	0.02162808	DLAT, FASN
**RES-MEL vs. ADQ-MEL**	Positive regulation of cellular component organization	0.01042504	TBC1D5,ATG2A,PLXNA1,PLXND1,EPHB2,MAP3K4,BCR,PLCG2,COLGALT1,SHOX2,KATNB1,SMPD3,PRKN,HTT,PIP4K2A,RREB1,ZNF205,CALCOCO2
	Cellular component organization	0.016238352	PXDN,TBC1D5,LAMP1,MAD1L1,CYP26C1,KIF26A,TUBGCP3,ATG2A,FILIP1,KCTD7,HDAC7,SLC12A7,PLXNA1,SZT2,TRIO,PLEKHF1,PLXND1,CTBP2,CAMSAP1,PPFIA1,EPHB2,MICAL3,AKT2,CDC42BPB,MAP3K4,OTOF,ATP9A,BCR,CAPZB,PLCG2,BRF1,PKP3,EHMT1,COLGALT1,KIRREL3,PLEC,SMARCA2,SHOX2,PRPF31,VAV2,SLC9A8,KATNB1,DSP,RAB11FIP3,LYRM4,SMPD3,NDUFA10,MYBL2,PRKN,SEC61A1,HTT,CSGALNACT1,KDM4A,MYO10,CAND2,PIP4K2A,VPS11,RREB1,RB1CC1,ZNF205,CHADL,SMARCB1,CALCOCO2,SPATA13
	Positive regulation of autophagy	0.025327935	ATG2A,PLEKHF1,PRKN,HTT,PIP4K2A,RB1CC1,CALCOCO2
	Catabolic process	0.046989112	LPCAT1,PXDN,TBC1D5,CYP26C1,IDUA,ATG2A,KLHL25,UBE2L3,PLEKHF1,EPHB2,UBE3C,AKT2,ZER1,PLCG2,PKP3,MKRN2,SMPD3,CTIF,PRKN,HTT,KDM4A,PIP4K2A,PLCL2,VPS11,RCN3,RB1CC1,GGA1,CALCOCO2,UBR3
	Cytoplasm	0.000129203	LPCAT1,PXDN,TBC1D5,LAMP1,MAD1L1,PPP1R12B,TUBGCP3,ATG2A,SLC41A3,KCTD7,TNKS1BP1,HDAC7,KLHL25,PLXNA1,SZT2,TRIO,PLEKHF1,EEF1AKMT2,CLPTM1L,CTBP2,TRAPPC9,CAMSAP1,PPFIA1,MICAL3,AKT2,CDC42BPB,PRKCA,TMC6,TMEM110,GMDS,MAP3K4,OTOF,ATP9A,CYTH3,BCR,CAPZB,PLCG2,EIF2AK1,MOB2,PKP3,FARS2,KLC1,MKRN2,COLGALT1,KIRREL3,PLEC,TAB1,VAC14,PTPN6,ADAP2,D2HGDH,VAV2,SLC9A8,KATNB1,DSP,RAB11FIP3,LYRM4,SMPD3,CTIF,INPP5E,NDUFA10,ADAP1,MYBL2,PRKN,SEC61A1,IRF8,HTT,AIP,CSGALNACT1,KDM4A,PTMA,PI4KA,MYO10,RPL3L,CAND2,PIP4K2A,VPS11,ERG,RCN3,RB1CC1,TULP4,GPR108,GGA1,CALCOCO2,SPATA13
	Autophagosome	0.000186907	TBC1D5,LAMP1,HTT,PIP4K2A,VPS11,RB1CC1,CALCOCO2

	Presynaptic active zone	0.001953171	TRIO,CTBP2,PPFIA1,OTOF,VPS11
	Presynapse	0.014410865	LAMP1,TRIO,CTBP2,PPFIA1,EPHB2,OTOF,KIRREL3,PRKN,HTT,VPS11
	Intracellular anatomical structure	0.017324179	CEP112,LPCAT1,PXDN,TBC1D5,BANP,LAMP1,MAD1L1,PPP1R12B,TUBGCP3,ATG2A,SLC41A3,LOC786836,FILIP1,KCTD7,GTF3C1,TNKS1BP1,HDAC7,NCOR2,KLHL25,PLXNA1,SZT2,TRIO,PLEKHF1,FAM53B,EEF1AKMT2,CLPTM1L,CTBP2,TRAPPC9,CAMSAP1,PPFIA1,CIC,EPHB2,MICAL3,AKT2,CDC42BPB,PRKCA,TMC6,TMEM110,GMDS,MAP3K4,OTOF,ATP9A,CYTH3,BCR,CAPZB,PLCG2,BRF1,EIF2AK1,ANKRD61,MAML3,MOB2,PKP3,FARS2,KLC1,EHMT1,MKRN2,COLGALT1,KIRREL3,PLEC,TAB1,SMARCA2,NUP210,VAC14,SHOX2,PRPF31,PTPN6,ADAP2,D2HGDH,VAV2,SLC9A8,KATNB1,DSP,RAB11FIP3,LYRM4,SMPD3,CTIF,H2B,INPP5E,NDUFA10,ADAP1,MYBL2,PRKN,SEC61A1,IRF8,HTT,AIP,CSGALNACT1,KDM4A,PTMA,PI4KA,MYO10,TCF20,RPL3L,CAND2,PIP4K2A,VPS11,ERG,RREB1,RCN3,RB1CC1,TULP4,GPR108,GGA1,SMARCB1,CALCOCO2,SPATA13
	Golgi apparatus subcompartment	0.030116412	TRAPPC9,ATP9A,SLC9A8,RAB11FIP3,SMPD3,CSGALNACT1,GPR108,GGA1
	Phosphatidylinositol-3,4,5-trisphosphate binding	0.033847491	CYTH3,ADAP2,ADAP1,MYO10
**MEL-MALE vs. CON-MALE**	Cytoplasm	4.23E-06	KIF5C,TUBGCP6,GRAMD4,TFDP1,BBC3,CAMSAP2,SEC16A,TELO2,NAA40,HDAC4,CTSD,CUL9,DFFB,ASAP2,SMPD5,LRBA,MAB21L2,KCNQ1,PRKCA,DGCR8,WDR7,LOC617396,CDC42BPG,XYLT2,CHKA,SNX8,CUL4A,TBC1D14,EEF1AKMT2,RNH1,PIEZO1,KLHL25,SELENOO,CNOT3,DNAJB6,ARL8A,PPFIA1,LETM1,PI4KA,PKD1,TULP3,NADSYN1,BCR,AFAP1,ADAD2,MYO18A,GRK5,PLEC,NOC2L,ABCA5,NFKBIE,MVB12B,BICDL1,USP12,GLI3,TBCD,NCOR1,GAB2,ERGIC1,KDM4B,EIPR1,GMDS,SUN1,KIF13B,TRRAP,P4HA2,PDLIM4,FGF14,IKBKE,CLIP2,LZTR1,SHANK2,GTF2IRD1,CYTH3,SIPA1L3,PACS2,FCHSD1,DNM1,GOLGA2,ZDHHC24,PPP6R1,CTIF,ACOX3,AFDN,PMVK,NPRL2,SCLY,AP1B1,HLCS,USP42,PEMT,ERGIC3,FGF18,LOC112443175,EDEM1,LAMP1,CAMSAP1,ERG,CAMK2A,ATP9B,VSIG10L,AXIN2,MAD1L1,PPP5C,IP6K2,DDAH2,MYOM3,CHDH,SPATA13,LIMK1,MICAL3,PCNT,TFEB
	Cytosol	0.000492967	TUBGCP6,TFDP1,CAMSAP2,SEC16A,TELO2,NAA40,HDAC4,CUL9,DFFB,ASAP2,LRBA,DGCR8,CDC42BPG,CHKA,SNX8,TBC1D14,EEF1AKMT2,RNH1,DNAJB6,BCR,AFAP1,GRK5,PLEC,NOC2L,NFKBIE,MVB12B,GLI3,TBCD,NCOR1,KDM4B,P4HA2,GTF2IRD1,CYTH3,PPP6R1,CTIF,AFDN,PMVK,SCLY,AP1B1,HLCS,USP42,PEMT,LAMP1,ERG,PPP5C,SPATA13,LIMK1,MICAL3,PCNT,TFEB
	Intracellular anatomical structure	0.019756628	KIF5C,TLE3,NR2C2,TUBGCP6,GRAMD4,MED15,TFDP1,BBC3,CAMSAP2,SEC16A,ZNF316,BOP1,SCX,TELO2,NAA40,HDAC4,NCAPH2,CTSD,H2B,CUL9,DFFB,ASAP2,SMPD5,MYO1H,DEAF1,LRBA,MAB21L2,KCNQ1,PRKCA,DGCR8,WDR7,LOC617396,CDC42BPG,XYLT2,CHKA,SNX8,CUL4A,TBC1D14,KMT5B,FAM53B,EEF1AKMT2,RNH1,CAMTA1,PIEZO1,NUP210,KLHL25,MAP2K5,SELENOO,CNOT3,DNAJB6,ARL8A,PPFIA

			1,LETM1,PI4KA,PKD1,TULP3,NADSYN1,BCR,AFAP1,TAF1C,ADAD2,MYO18A,GRK5,PLEC,NOC2L,CEP164,ABCA5,NFKBIE,MVB12B,BICDL1,USP12,GLI3,TBCD,NCOR1,GAB2,ERGIC1,KDM4B,EIPR1,GMDS,SUN1,KIF13B,TRRAP,P4HA2,PDLIM4,FGF14,IKBKE,CLIP2,LZTR1,SHANK2,GTF2IRD1,CYTH3,SIPA1L3,NFATC1,PACS2,FCHSD1,DNM1,GOLGA2,ZDHHC24,PPP6R1,CTIF,ACOX3,AFDN,CHD7,NSD3,PMVK,NPRL2,HR,PRAG1,SCLY,AP1B1,HLCS,USP42,PEMT,ERGIC3,GTF3C1,FGF18,AFF1,LOC112443175,EDEM1,LAMP1,CAMSAP1,ERG,CAMK2A,AHDC1,ATP9B,VSIG10L,AXIN2,MAD1L1,PPP5C,IP6K2,DDAH2,MYOM3,CHDH,SPATA13,LIMK1,MICAL3,L3MBTL2,PCNT,TFEB
	Nucleoplasm	0.038994372	TLE3,NR2C2,MED15,TFDP1,BOP1,TELO2,NAA40,HDAC4,NCAPH2,DFFB,DEAF1,DGCR8,TBC1D14,KMT5B,EEF1AKMT2,RNH1,DNAJB6,TULP3,TAF1C,GRK5,NOC2L,CEP164,NFKBIE,GLI3,NCOR1,KDM4B,TRRAP,P4HA2,IKBKE,GTF2IRD1,CYTH3,SIPA1L3,NFATC1,AFDN,CHD7,HR,GTF3C1,AFF1,ERG,VSIG10L,IP6K2,SPATA13,LIMK1,MICAL3
	Protein binding	0.000315171	DGCR2,KIF5C,TLE3,TUBGCP6,TFDP1,PTPRF,LRP5,CAMSAP2,BOP1,SCX,TELO2,HDAC4,H2B,CUL9,CUL1,DFFB,ASB13,ASAP2,MYO1H,LRBA,OLFML2A,TBC1D22A,PPP4R1,KCNQ1,DGCR8,WDR7,CHKA,SNX8,CUL4A,TBC1D14,KMT5B,HSPG2,RNH1,CAMTA1,KLHL25,MAP2K5,DNAJB6,PPFIA1,PKD1,TULP3,AFAP1,PLCL2,TAF1C,MYO18A,JAG2,PLEC,NOC2L,CEP164,NFKBIE,TTC21A,GLI3,TBCD,NCOR1,GAB2,KLHDC4,PPP1R37,EIPR1,FLRT2,GMDS,SUN1,KIF13B,TRRAP,P4HA2,PDLIM4,FGF14,IKBKE,CLIP2,LZTR1,SHANK2,SIPA1L3,LRP4,PACS2,FCHSD1,DNM1,GOLGA2,PPP6R1,CTIF,AFDN,CHD7,NSD3,PRAG1,AP1B1,HLCS,FGF18,LOC112443175,EDEM1,LAMP1,CAMSAP1,ERG,CAMK2A,AXIN2,MAD1L1,PPP5C,MYOM3,SPATA13,LIMK1,MICAL3,L3MBTL2,TFEB
**MEL-FEMALE vs. CON-FEMALE**	positive regulation of biological process	0.024738536	RREB1,GPER1,YTHDF1,ZBED4,DGKQ,ADARB1,LRRC8A,MAD1L1,STUB1,JMJD8,PLXNA1,HTT,EXOSC10,PLXND1,HIP1R,NF2,KLHL25,RPTOR,LIMK1,EIPR1,ZSCAN2,ZMYND8,BICRA,SART1,PLAG1,SMOC2,SLC6A6,LRSAM1,SS18L1,CDT1,NEK2,BEND3,ZFP64,CAMK4,SLC45A3,EHMT1,NEK7,BCR,TRAPPC12,WHRN,EVL,WWOX,MEGF8,TBC1D5,CYTH3,PKDCC,DEF8,IKBKG,CUL4A,SIK1,ZNRF1,PTMA,IKBKB,TPPP,AMIGO1,NOC2L,TMEM161A,ZFPM1,CDK5R1,NEDD4L,SHANK2,LRP2,ATP10A,JAG2,MARK4,UBE2E2,KCNQ1,CAPN10,CDH4,SPIDR,ARIH2,HDAC4,YBX3,ZBED1,DHRSX,MCF2L,EPHA2,MED27,BOLA,NFIA,PIP4K2A,PAXIP1,SLC20A2,NBAS,MAML3,SLC24A1,POLR3A,LAMC2,PREX1,PELP1,SNX9,MTA3,EPHB1,ASS1,LAMP1,SUPT5H,CNST,RPS6KA5,RBM19,GPC1,FRMD1,VAV2,LIMD1,MYLIP,WASF2,ABCA2,FOXJ1,SREBF2,MAN2A1,TENM4,PPARGC1B,RNF40,SETD4,BRD1,ITM2C,PRKCD,TNIK,RARB,MFSD2A,NFATC2,ECSCR,CASP3,MTF1
	actin filament-based process	0.050235479	STARD13,PLEC,FHOD3,SPECC1L,CELSR1,HIP1R,NF2,LIMK1,CDC42BPB,FAT1,SHROOM2,BCR,EVL,IKBKB,CDK5R1,KANK2,CACNA1C,SMTNL1,KCNQ1,CAPN10,ARPC1B,MYO9B,LLGL2,NISCH,SNX9,ELMO2,WASF2,FOXJ1,EPB41L1,PPARGC1B,PRKCD,TNIK

	cell development	0.030651341	CFAP73,GPER1,RAB17,PLEC,ADARB1,TRIO,FHOD3,LRRC8A,PLXNA1,PLXND1,CAMSAP1,NF2,KLHL25,LIMK1,MYT1L,SART1,PLAG1,SS18L1,FAT1,CAMK4,BCR,WHRN,MEGF8,CUL4A,IKBKB,TPPP,ZFPM1,CDK5R1,NEDD4L,LRP2,JAG2,KCNQ1,CDH4,ARIH2,HDAC4,YBX3,EPHA2,PIP4K2A,CDHR1,MAP4K4,PREX1,EPHB1,TRIM10,DOCK1,ACRBP,LIMD1,MYLIP,WASF2,NTN5,FOXJ1,MAN2A1,TENM4,CNTRL,PPARGC1B,BRD1,ITM2C,NTNG2,TNIK,RARB,MFSD2A,SCYL1,NFATC2,CASP3,PTPRF
	multicellular organism development	0.026107363	STARD13,GPER1,GREB1L,RAB17,PLEC,ADARB1,TRIO,FHOD3,MAD1L1,JMJD8,PLXNA1,PLXND1,CELSR1,CAMSAP1,HIP1R,NF2,KLHL25,LIMK1,MYT1L,ZMYND8,RTTN,SART1,PLAG1,SMOC2,SS18L1,NEK2,TRAPPC9,FAT1,SLC45A3,EHMT1,BCR,WDR37,MMP15,WHRN,WWOX,MEGF8,PKDCC,CUL4A,ADAMTS16,SIK1,IKBKB,TPPP,AMIGO1,ZFPM1,JARID2,CDK5R1,KANK2,NEDD4L,LRP2,JAG2,CACNA1C,MARK4,EXT2,KCNQ1,CDH4,HDAC4,YBX3,EPHA2,NFIA,PAXIP1,SLC20A2,CDHR1,SGMS2,MAP4K4,EPHB1,EPHB4,RBM19,MYLIP,WASF2,NTN5,FOXJ1,MAN2A1,TENM4,CNTRL,PPARGC1B,CLUAP1,MBD3,BRD1,ITM2C,NTNG2,TNIK,RARB,MFSD2A,SCYL1,NFATC2,ECSCR,CASP3,MTF1,PTPRF
	system development	0.026508546	STARD13,GPER1,GREB1L,RAB17,PLEC,ADARB1,TRIO,FHOD3,MAD1L1,JMJD8,PLXNA1,PLXND1,CELSR1,CAMSAP1,HIP1R,NF2,LIMK1,MYT1L,ZMYND8,PLAG1,SMOC2,SS18L1,TRAPPC9,FAT1,SLC45A3,BCR,WDR37,WHRN,WWOX,MEGF8,PKDCC,ADAMTS16,SIK1,TPPP,AMIGO1,ZFPM1,JARID2,CDK5R1,KANK2,NEDD4L,LRP2,JAG2,CACNA1C,MARK4,EXT2,KCNQ1,CDH4,HDAC4,YBX3,EPHA2,NFIA,PAXIP1,CDHR1,MAP4K4,EPHB1,EPHB4,MYLIP,WASF2,NTN5,FOXJ1,MAN2A1,TENM4,CNTRL,PPARGC1B,CLUAP1,ITM2C,NTNG2,TNIK,RARB,MFSD2A,SCYL1,NFATC2,ECSCR,CASP3,MTF1,PTPRF
	cell differentiation	0.02581262	RREB1,CFAP73,GPER1,RAB17,PLEC,ADARB1,TRIO,FHOD3,LRRC8A,PLXNA1,PLXND1,CELSR1,CAMSAP1,NF2,KLHL25,LIMK1,MYT1L,SART1,PLAG1,SLC6A6,SS18L1,TRAPPC9,ZFP64,FAT1,CAMK4,SLC45A3,BCR,MMP15,WHRN,WWOX,MEGF8,UNC45A,PKDCC,CUL4A,SIK1,IKBKB,TPPP,ZFPM1,JARID2,CDK5R1,NEDD4L,LRP2,JAG2,EXT2,KCNQ1,CDH4,ARIH2,HDAC4,YBX3,EPHA2,NFIA,PIP4K2A,PAXIP1,CDHR1,MAP4K4,PREX1,EPHB1,TRIM10,DOCK1,GPC1,ACRBP,LIMD1,MYLIP,WASF2,NTN5,FOXJ1,MAN2A1,TENM4,CNTRL,PPARGC1B,BRD1,ITM2C,NTNG2,TNIK,SPATA20,RARB,MFSD2A,SCYL1,NFATC2,CASP3,PTPRF
	tissue development	0.031209363	RREB1,STARD13,GREB1L,PLEC,ADARB1,FHOD3,PLXNA1,PLXND1,TFIP11,CELSR1,NF2,ZFP64,FAT1,BCR,MMP15,WHRN,MEGF8,PKDCC,ADAMTS16,SIK1,IKBKB,ZFPM1,JARID2,KANK2,LRP2,JAG2,EXT2,KCNQ1,HDAC4,YBX3,EPHA2,NFIA,PAXIP1,SLC20A2,SGMS2,LAMC2,EPHB1,GPC1,NTN5,FOXJ1,TENM4,CLUAP1,MBD3,NTNG2,RARB,MFSD2A,NFATC2,CASP3

	intracellular signal transduction	0.027857829	PI4KA,STARD13,GPER1,DGKQ,MAD1L1,JMJD8,HTT,CELSR1,NF2,RPTOR,MGRN1,CDT1,CAMK4,BCR,WWOX,CYTH3,IKBKG,CUL4A,SIK1,IKBKB,NOC2L,TMEM161A,KANK2,SHANK2,LRP2,MARK4,PKN3,ARFGAP1,HDAC4,YBX3,MCF2L,MAP3K6,EPHA2,ARHGEF12,MYO9B,PAXIP1,DDIT4,MAP4K4,PSCA,NISCH,MAPK1,PREX1,EPHB1,RPS6KA5,DOCK1,OTUD3,TPCN2,FRMD1,MBTPS1,VAV2,LIMD1,WASF2,RALGPS1,MAP3K9,PRKCD,TNIK,NFATC2,CASP3
	regulation of cellular component biogenesis	0.037958115	RAB17,FHOD3,HTT,TFIP11,CAMSAP1,HIP1R,NF2,LIMK1,LRSAM1,NEK7,TRAPPC12,EVL,DEF8,IKBKB,TPPP,AMIGO1,CDK5R1,KANK2,MARK4,SPIDR,TBC1D14,EPHA2,PIP4K2A,SNX9,EPHB1,WASF2,ABCA2,NTNG2,PRKCD
	positive regulation of nervous system development	0.066326531	GPER1,PLXNA1,PLXND1,LIMK1,PLAG1,SS18L1,MEGF8,AMIGO1,LRP2,CDH4,EPHB1,MAN2A1,TENM4<br>GPER1,RAB17,PLEC,ADARB1,TRIO,PLXNA1,PLXND1,CELSR1,CAMSAP1,NF2,LIMK1,MYT1L,ZMYND8,PLAG1,SS18L1,TRAPPC9,SLC45A3,BCR,WDR37,WHRN,MEGF8,TPPP,AMIGO1,CDK5R1,NEDD4L,LRP2,JAG2,MARK4,KCNQ1,CDH4,EPHA2,NFIA,CDHR1,MAP4K4,EPHB1,MYLIP,NTN5,FOXJ1,MAN2A1,TENM4,CLUAP1,ITM2C,NTNG2,TNIK,RARB,MFSD2A,SCYL1,CASP3,MTF1,PTPRF
	nervous system development	0.02895194	TRIO,GRK4,LIMK1,NEK2,CDC42BPB,CAMK4,NEK7,BCR,SIK1,IKBKB,MARK4,PKN3,MAP3K6,MAP4K4,MAPK1,RPS6KA5,MAP3K9,PRKCA,PRKCD,TNIK
	protein serine/threonine kinase activity	0.051813472	DDX59,EEFSEC,DGKQ,RAB17,MYO1H,TRIO,PLXNA1,GRK4,PLXND1,FARS2,LIMK1,NEK2,CDC42BPB,CAMK4,NEK7,LIG1,EHD4,PKDCC,UCK1,SIK1,IKBKB,DNAH17,PGS1,KIF25,NADSYN1,ATP10A,MARK4,UBE2E2,PKN3,ATP9B,MAP3K6,EPHA2,PIP4K2A,MYO9B,ZRANB3,CARNS1,MAP4K4,MAPK1,EPHB1,ASS1,EPHB4,RPS6KA5,ABCA2,RECQL5,MAP3K9,ITM2C,PRKCA,PRKCD,TNIK,ERAL1,SCYL1
	purine ribonucleoside triphosphate binding	0.029912023	AP2A1,TRIO,PLXNA1,PLXND1,LIMK1,CDK5R1,EPHA2,ARHGEF12,ARPC1B,MYO9B,MAPK1,AP2A2,EPHB1,EPHB4,RPS6KA5,DOCK1,GPC1,VAV2
	Axon guidance	0.090909091	AP2A1,TRIO,PLXNA1,PLXND1,LIMK1,CDK5R1,EPHA2,ARHGEF12,ARPC1B,MYO9B,MAPK1,AP2A2,EPHB1,EPHB4,RPS6KA5,DOCK1,GPC1,VAV2
	Nervous system development	0.090452261	NF2,LIMK1,ARPC1B,MAPK1,DOCK1,VAV2,ELMO2,WASF2,PRKCD
	Fcgamma receptor (FCGR) dependent phagocytosis	0.140625	STARD13,BCR,PKN3,MCF2L,ARHGEF12,MYO9B,PREX1,VAV2
	RHOC GTPase cycle Developmental Biology	0.126984127	AP2A1,TRIO,PLXNA1,PLXND1,LIMK1,CDK5R1,NCOR2,CDH4,EPHA2,ARHGEF12,ARPC1B,MYO9B,MAPK1,AP2A2,EPHB1,EPHB4,RPS6KA5,DOCK1,GPC1,VAV2

	RHOG GTPase cycle	0.056657224	TRIO,MCF2L,EPHA2,LETM1,PREX1,DOCK1,VAV2,ELMO2
	Signaling by Rho GTPases	0.045528455	STARD13,TRIO,MAD1L1,PLXNA1,PLXND1,NF2,LIMK1,CDC42BPB,BCR,H2B,KLC4,UBXN11,PKN3,MCF2L,EPHA2,ARHGEF12,ARPC1B,MYO9B,LETM1,NISCH,MAPK1,PREX1,DOCK1,VAV2,ELMO2,WASF2,PRKCD,CENPM
	BDNF signaling pathway	0.08	CAMK4,IKBKG,IKBKB,CDK5R1,MAPK1,RPS6KA5,VAV2,PRKCD,CASP3,PTPRF

Table S8. Functional enrichment of significant DMRs across different comparisons
